# A CARMIL2 gain-of-function mutation suffices to trigger most CD28 costimulatory functions in vivo

**DOI:** 10.1084/jem.20250339

**Published:** 2025-05-22

**Authors:** Fanghui Zhang, Javier Celis-Gutierrez, Lichen Zhang, Valentin Mellado, Léna Gelard, Sophie Panigot, Daiki Mori, Liaoxun Lu, Guillaume Voisinne, Carine Vilarnau Wolek, Marielle Mello, Odile Burlet-Schiltz, Anne Gonzalez de Peredo, Frédéric Fiore, Romain Roncagalli, Yinming Liang, Marie Malissen, Bernard Malissen

**Affiliations:** 1 Centre d’Immunologie de Marseille-Luminy (CIML), Aix Marseille Université, Institut national de la santé et de la recherche médicale (INSERM), Centre national de la recherche scientifique (CNRS), Marseille, France; 2 School of Medical Technology, Xinxiang Medical University, Xinxiang City, China; 3 Centre d’Immunophénomique (CIPHE), Aix Marseille Université, INSERM, CNRS, Marseille, France; 4 Institut de Pharmacologie et de Biologie Structurale (IPBS), Université de Toulouse, CNRS, Université Toulouse III - Paul Sabatier (UPS), Toulouse, France; 5 Laboratory of Mouse Genetics, Institute of Psychiatry and Neuroscience, Xinxiang Medical University, Xinxiang City, China; 6 Laboratory of Immunophenomics, School of Medical Technology, Xinxiang Medical University, Xinxiang City, China

## Abstract

Naive T cell activation requires both TCR and CD28 signals. The CARMIL2 cytosolic protein enables CD28-dependent activation of the NF-κB transcription factor via its ability to link CD28 to the CARD11 adaptor protein. Here, we developed mice expressing a mutation named *Carmil2*^QE^ and mimicking a mutation found in human T cell malignancies. Naive T cells from *Carmil2*^QE^ mice contained preformed CARMIL2^QE^-CARD11 complexes in numbers comparable to those assembling in wild-type T cells after CD28 engagement. Such ready-made CARMIL2^QE^-CARD11 complexes also formed in CD28-deficient mice where they unexpectedly induced most of the functions that normally result from CD28 engagement in a manner that remains antigen-dependent. In turn, tumor-specific T cells expressing *Carmil2*^QE^ do not require CD28 engagement and thereby escape to both PD-1 and CTLA-4 inhibition. In conclusion, we uncovered the overarching role played by CARMIL2-CARD11 signals among those triggered by CD28 and exploited them to induce potent solid tumor–specific T cell responses in the absence of CD28 ligands and immune checkpoint inhibitors.

## Teaser

Zhang et al. demonstrate that the expression of a mutated CARMIL2 protein in CD28-deficient mice induces most of the developmental and functional consequences known to result from CD28 costimulation and in turn triggers potent tumor-specific T cell responses resistant to PD-1 and CTLA-4 blockade.

## Introduction

Although essential, TCR signals are not sufficient for the optimal activation of naive T cells. Concomitant CD28 signals are also required to promote the activation and differentiation of naive T cells into effector and memory T cells and to control peripheral tolerance by contributing to the development and function of regulatory T (T_reg_) cells (Carter and Pomerantz, 2022; Vang et al., 2010). The importance of CD28 costimulation has been demonstrated by the failure of CD28-deficient mice to produce T cell–dependent antibody responses and to clear certain infections (Ferguson et al., 1996; McSorley and Jenkins, 2000; Shahinian et al., 1993; Welten et al., 2015). These findings have led to the “two-signal model” of naive T cell activation in which signal 1 is delivered by the TCR after recognition of antigenic peptides bound to MHC molecules and signal 2 is provided by CD28 after binding to its CD80 (B7.1) and CD86 (B7.2) ligands expressed on immunogenic antigen-presenting cells (APC) (Burke et al., 2024). CD28 costimulation also plays a crucial role during the activation of antigen-experienced T cells such as tumor-specific effector T cells (Agarwal et al., 2023; Duraiswamy et al., 2021; Kamphorst et al., 2017; Magen et al., 2023), and the rescue of exhausted CD8^+^ T cells by PD-1–targeted therapies (Kamphorst et al., 2017). The protein–protein interaction motifs found in the CD28 cytoplasmic tail trigger multiple signaling pathways. Their respective contributions to the developmental and functional outcomes resulting from CD28 costimulation remain, however, controversial (Pagán et al., 2012).

A loss-of-function mutation in the mouse *Carmil2* gene demonstrated that the capping protein regulator and myosin 1 linker 2 (CARMIL2) protein it encodes is dispensable for the delivery of TCR signals but essential for CD28 costimulation (Liang et al., 2013). CARMIL2 (also known as RLTPR) is a multidomain cytosolic protein that participates in the CD28 microclusters that form at the immunological synapse where it connects CD28 to the CARD11 adaptor protein (Liang et al., 2013). It allows subsequent phosphorylation of the recruited CARD11 molecules (also known as CARMA1) by TCR-activated protein serine/threonine kinases and in turn leads to the assembly of CARD11-BCL10-MALT1 (CBM) complexes capable of activating the NF-κB transcription factor, the c-Jun N-terminal kinase, and the MALT1 protease (Roncagalli et al., 2016; Ruland and Hartjes, 2019). The scaffolding function of CARMIL2 was further confirmed by the identification of both CD28 and CARD11 in the set of proteins that associate with CARMIL2 following CD28 engagement and form the CARMIL2 interactome (Liu et al., 2018; Roncagalli et al., 2016).

T cells from *CARMIL2*-deficient patients also showed normal TCR-mediated signals and defective CD28-mediated NF-κB activation (Lévy et al., 2023; Schober et al., 2017; Wang et al., 2016), suggesting that CARMIL2 molecules exert overlapping functions in mouse and human T cells. A *CARMIL2* point mutation denoted as *CARMIL2* p.Q575E and corresponding to a nonconservative substitution of the glutamine residue found at position 575 by a glutamic acid has been identified in 3% of human cutaneous T cell lymphoma and acute T cell leukemia (Park et al., 2017; Uchida et al., 2021). When overexpressed in human Jurkat leukemic T cells, CARMIL2^Q575E^ molecules constitutively associated with CARD11 molecules, leading to augmented NF-κB activation and interleukin 2 (IL-2) mRNA expression following stimulation with pharmacologic TCR mimics (Park et al., 2017; Uchida et al., 2021). Here, we developed gene-edited mice expressing a mutation orthologous to *CARMIL2* p.Q575E and denoted as *Carmil*2^Q538E^ (in short *Carmil*2^QE^), and we determined its effects on the development and function of mouse T cells in vivo. Naive T cells from *Carmil*2^QE^ mice contained preformed CARMIL2^QE^-CARD11 complexes in numbers comparable to those that assemble in naive wild-type (WT) T cells after CD28 engagement. Considering that those preformed CARMIL2^QE^-CARD11 complexes can be generated irrespective of CD28 expression, we surmised that their phosphorylation by TCR-operated serine/threonine kinases should generate functional CBM complexes in the absence of CD28 engagement. Consistent with this hypothesis, when expressed in CD28-deficient mice, those “ready-made” CARMIL2^QE^-CARD11 complexes induced proper thymic T_reg_ cell development, an outcome known to specifically require CD28-CARMIL2-CARD11 signals (Carter and Pomerantz, 2022; Liang et al., 2013; Tai et al., 2005; Vang et al., 2010). It provided the unique opportunity to assess the importance of the CARMIL2-CARD11 signaling branch among the multiple signaling branches triggered by CD28. Unexpectedly, the expression of the *Carmil2*^QE^ mutation in CD28-deficient mice triggered most of the known developmental and functional consequences resulting from CD28 costimulation in vivo and it occurred in a manner that remained dependent on TCR signals. Therefore, our results uncover the overarching role of CARMIL2-CARD11–driven signals among those triggered by CD28 in vivo and we demonstrated that they can be exploited to generate potent tumor-specific CD8^+^ T cells that do not require CD28 engagement and thereby escape to both PD-1 and CTLA-4 inhibition.

## Results

### Effects of the *CARMIL2*^QE^ mutation on physiological Jurkat T cell activation

The mouse *Carmil2* gene gives rise to several transcripts via alternative splicing. We originally described a mouse *Carmil2* transcript sequence coding for functional CARMIL2 proteins (Liang et al., 2013; Roncagalli et al., 2016). Its human ortholog is denoted as isoform 3 and also codes for functional CARMIL2 proteins (Lévy et al., 2023; Park et al., 2017; Uchida et al., 2021). The human *CARMIL2* Q575E mutation has been numbered on the basis of human isoform 1, which codes for nonfunctional CARMIL2 proteins due to an adventitious insertion into exon 14 (Lévy et al., 2023; Park et al., 2017; Uchida et al., 2021). To conform to the functional isoform 3 amino acid sequence, the human *CARMIL2* Q575E mutation has been renumbered here as *CARMIL2* Q539E (abbreviated as *CARMIL2*^Q539E^ or *CARMIL2*^QE^) and its mouse counterpart as *Carmil2* Q538E (abbreviated as *Carmil2*^Q538E^ or *Carmil2*^QE^).

**Figure 1.**
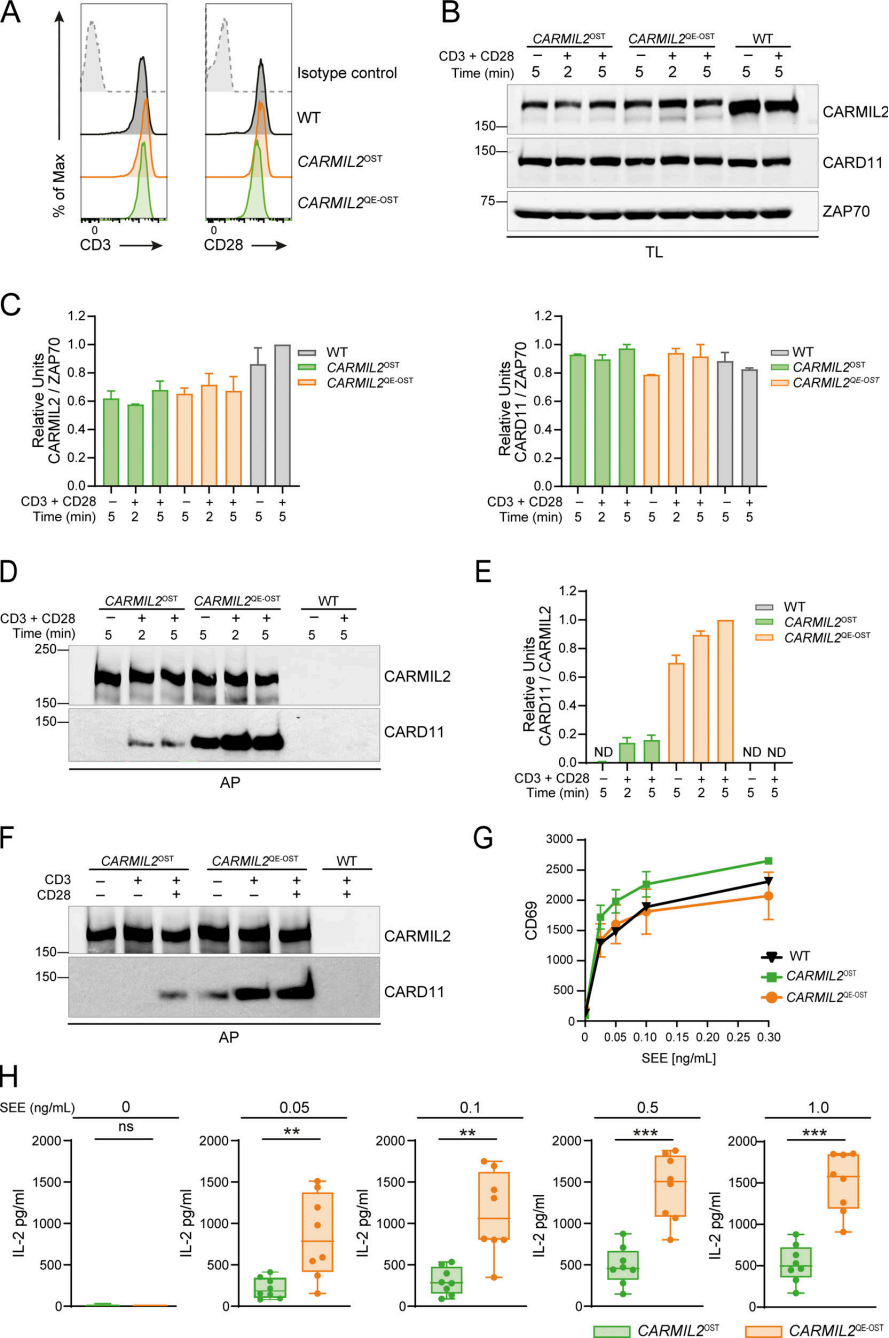
**Effects of the *CARMIL2*** ^**QE**^ **mutation on physiological Jurkat T cell activation. (A)** WT, *CARMIL2*^OST^, and *CARMIL2*^QE-OST^ Jurkat T cells were analyzed by flow cytometry for the expression of CD3 and CD28 (shaded histograms). Dashed line curves correspond to isotype-matched control antibodies (negative controls), and data are representative of two independent experiments. **(B)** WT, *CARMIL2*^OST^, and *CARMIL2*^QE-OST^ Jurkat T cells were left untreated (−) or stimulated (+) with anti-CD3 plus anti-CD28 for 2 and 5 min at 37°C. Immunoblot analysis of equal amounts of TL of the specified cells probed with anti-CARMIL2, anti-CARD11, and an anti-ZAP70 loading control. Data are representative of two independent experiments. Left margin, molecular size in kilodaltons. **(C)** Quantitation of the immunoblot analysis shown in B. Bars represent normalized CARMIL2-ZAP-70 and CARD11-ZAP70 ratios (see Materials and methods). Data in C and E are presented as the mean ± SE. **(D)** WT, *CARMIL2*^OST^, and *CARMIL2*^QE-OST^ Jurkat T cells were activated as in B, and immunoblot analysis of equal amounts of lysates from the specified cells subjected to AP on Strep-Tactin Sepharose beads, followed by elution of proteins with D-biotin, and probed with anti-CARMIL2 or anti-CARD11. Data are representative of two independent experiments. Left margin, molecular size in kilodaltons. **(E)** Quantitation of the immunoblot analysis shown in D. Bars represent normalized CARD11-CARMIL2 ratios. **(F)***CARMIL2*^OST^ and *CARMIL2*^QE-OST^ Jurkat T cells were left untreated (−) or stimulated with anti-CD3 (+) in the presence (+) or absence (−) of anti-CD28 for 2 min at 37°C. Immunoblot analysis of equal amounts of lysates from the specified cells subjected to AP on Strep-Tactin Sepharose beads, followed by elution of proteins with D-biotin, and probed with anti-CARMIL2 or anti-CARD11. Data are representative of two independent experiments. Left margin, molecular size in kilodaltons. **(G)** WT, *CARMIL2*^OST^, and *CARMIL2*^QE-OST^ Jurkat cells were stimulated with Raji cells that were preincubated in the absence (0) or presence of the specified concentrations of SEE. For each condition, the MFI of CD69^+^ cells was measured by flow cytometry 24 h after stimulation. Numbers on the y axis correspond to the MFI of CD69^+^ cells. Error bars correspond to the mean and SD. Data are representative of two independent experiments. **(H)** IL-2 production by *CARMIL2*^OST^ and *CARMIL*2^QE-OST^ Jurkat T cell clones stimulated with Raji cells alone (0) or in the presence of 0.05, 0.1, and 0.5 and 1 ng/ml SEE. Analysis of IL-2 production was performed 24 h after stimulation. The expression of IL-2 (pg/ml) is shown using boxplot with the median, boxed interquartile range, and whiskers extending to the most extreme point up to 1.5 times the interquartile range. Data are representative of two independent experiments, involving eight independent clones of *Carmil2*^OST^ and *Carmil2*^Q539E- OST^ Jurkat cells. Each dot corresponds to a clone of the specified Jurkat T cells. **P < 0.01, ***P ≤ 0.001, and ns, nonsignificant; unpaired Student’s *t* test. AP, affinity purification; TL, total lysates; MFI, mean fluorescence intensity. Source data are available for this figure: SourceData F1.

To assess the effects of the *CARMIL2*^QE^ mutation on human T cells stimulated with physiological triggers, Jurkat T cells were stimulated with Raji lymphoblastoid B cells that present the superantigen staphylococcal enterotoxin E (SEE) and express CD28 ligands. In this model, IL-2 production requires both TCR and CARMIL2-mediated CD28 signals, whereas CD69 expression only requires TCR signals and in turn can occur in the absence of CARMIL2 expression (Roncagalli et al., 2016; Tian et al., 2015). To facilitate biochemical analysis, the two endogenous copies of the Jurkat *CARMIL*2 gene were edited to produce CARMIL2 or CARMIL2^QE^ proteins tagged with an affinity Twin-Strep-tag (OST) and it did not affect CD3 and CD28 surface levels (Fig. 1 A). The resulting CARMIL2^OST^ and CARMIL2^QE-OST^ proteins were expressed at 1.5-fold lower levels than WT CARMIL2 proteins (Fig. 1, B and C). Affinity purification and immunoblot analysis showed that the association between CARMIL2^OST^ and CARD11 required prior TCR-CD28 stimulation, whereas CARMIL2^QE-OST^ molecules were already associated with CARD11 in the absence of TCR-CD28 stimulation (Fig. 1, D and E). The levels of CARMIL2^QE-OST^-CARD11 complexes present prior to activation further increased 1.5-fold following TCR-CD28 engagement. Importantly, stimulation of *CARMIL2*^OST^ Jurkat T cells with anti-CD3 alone failed to trigger the assembly of CARMIL2-CARD11 complexes (Fig. 1 F), demonstrating the specific contribution of CD28 engagement to the assembly of CARMIL2-CARD11 complexes in WT T cells. After stimulation with Raji B cells in the presence of SEE, *CARMIL2*^OST^ and *CARMIL2*^QE-OST^ Jurkat cells expressed levels of CD69 comparable to WT Jurkat cells (Fig. 1 G), whereas IL-2 production by *CARMIL2*^QE-OST^ Jurkat T cells was increased threefold as compared to WT and *CARMIL2*^OST^ Jurkat cells (Fig. 1 H). Therefore, congruent with former studies using Jurkat cells overexpressing CARMIL2^QE^ molecules and pharmacological mimics of TCR stimulation (Park et al., 2017; Uchida et al., 2021), human CARMIL2^QE^ proteins expressed at close to physiological levels induced the formation of CARMIL2^QE^-CARD11 complexes in unstimulated Jurkat cells resulting in a specific increase in IL-2 production following physiological TCR-CD28 stimulation.

### Resting *Carmil2*^QE^ mouse T cells contain preformed CARMIL2^QE^-CARD11 complexes

**Figure S1.**
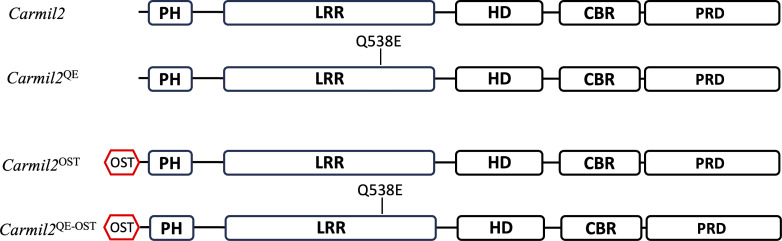
**Schematic structure of the CARMIL proteins produced by WT mice and *Carmil2*** ^**QE**^ **, *Carmil2*** ^**OST**^ **, and *Carmil2*** ^**QE-OST**^ **gene-targeted mice.** Mouse CARMIL2 proteins are 1,397–amino acid-long multidomain cytosolic proteins that consist of PH, LRR, HD, CBR, MBD, and PRD domains (Stark et al., 2017; Zwolak et al., 2013). The DNA strand opposite to the one coding for the 3′ untranslated region of the mouse *Carmil2* gene corresponds to the 3′ end of the *Acd* gene, which codes for a protein involved in telomere function, and its ablation is recessive lethal. In a former study, we developed gene-targeted mice expressing CARMIL2 proteins tagged at their carboxyl terminus with an affinity OST tag. The introduction of the OST coding sequence at the 3′ end of the *Carmil2* gene adventitiously impaired the expression of the *Acd* gene. It prevented the establishment of mice homozygous for the *Carmil2*^OST^ allele and reduced the sensitivity of our AP-MS analysis since only half of the CARMIL2 molecules were OST-tagged in viable heterozygous mice (Roncagalli et al., 2016). Therefore, to bypass this limitation, the present study uses mice in which the CARMIL2 and CARMIL2^QE^ proteins were tagged with an OST tag at their N terminus. As a result, mice homozygous for those *Carmil2*^OST^ and *Carmil2*^QE-OST^ alleles were born at expected Mendelian frequencies. Moreover, introduction of the OST tag at the N terminus of CARMIL2 and CARMIL2^QE^ molecules did not change their levels of expression as compared to their untagged counterparts (Fig. 2 A). *Carmil2*^QE^ mice expressing CARMIL2^QE^ molecules lacking an OST tag were also developed (see Materials and methods). They had a phenotype similar to that of *Carmil2*^QE-OST^ mice (Fig. S5) and were used interchangeably with *Carmil2*^QE-OST^ mice. PH, pleckstrin homology domain; LRR, leucine-rich region; HD, helical dimerization domain; CBR, capping protein-binding region; MBD, membrane-binding domain; PRD, proline-rich domain.

**Figure 2.**
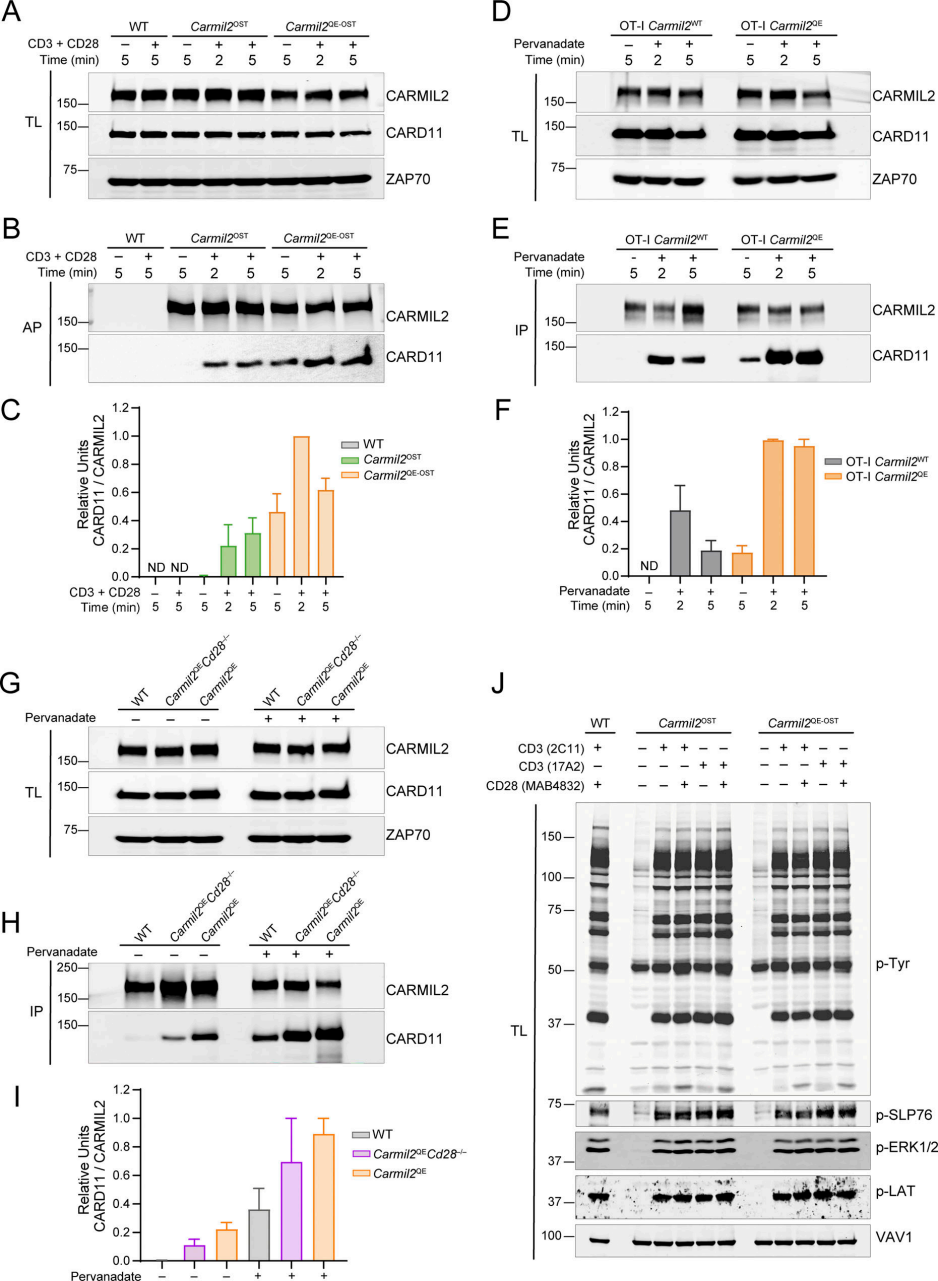
**Preformed CARMIL2** ^**QE**^ **-CARD11 complexes are found in unstimulated *Carmil2*** ^**QE**^ **mouse T cells irrespective of CD28 expression. (A)** CD4^+^ T cells purified from WT, *Carmil2*^OST^, and *Carmil2*^QE-OST^ mice were either left untreated (−) or stimulated (+) with anti-CD3 plus anti-CD28 for 2 and 5 min at 37°C. Equal amounts of TL of the specified mouse CD4^+^ T cells were analyzed by immunoblots and probed with anti-CARMIL2, anti-CARD11, and an anti-ZAP70 loading control. **(B)** Immunoblot analysis of equal amounts of lysates from mouse CD4^+^ T cells prepared as in A and subjected to AP on Strep-Tactin Sepharose beads, followed by elution of proteins with D-biotin, and probed with anti-CARMIL2 or anti-CARD11. **(C)** Quantitation of the immunoblot analysis shown in B. Bars represent normalized CARMIL2-ZAP70 and CARD11-ZAP70 ratios. Data are presented as the mean ± SE in C, F, and I. **(D)** CD8^+^ T cells purified from OT-I *Carmil*2^WT^ and OT-I *Carmil*2^QE^ mice were either left untreated (−) or stimulated (+) with pervanadate for 2 and 5 min at 37°C. Equal amounts of TL of the specified mouse CD8^+^ T cells were analyzed by immunoblots and probed with anti-CARMIL2, anti-CARD11, and an anti-ZAP70 loading control. **(E)** Immunoblot analysis of equal amounts of lysates from mouse CD8^+^ T cells activated as in D and from which CARMIL2 or CARMIL2^QE^ proteins were IP with an anti-CARMIL2 antibody, and subjected to immunoblot analysis with anti-CARMIL2 or CARD11. **(F)** Quantitation of the immunoblot analysis shown in E. Bars represent normalized CARMIL2-ZAP70 and CARD11-ZAP70 ratios. **(G)** CD4^+^ T cells purified from WT, *Carmil2*^QE^, and *Carmil2*^QE^*Cd28*^−/−^ mice were either left untreated (−) for 2 min at 37°C or stimulated (+) with pervanadate for 2 min at 37°C. Equal amounts of TL of the specified mouse CD4^+^ T cells were analyzed by immunoblots and probed with anti-CARMIL2, anti-CARD11, and an anti-ZAP70 loading control. **(H)** Immunoblot analysis of equal amounts of lysates from mouse CD4^+^ T cells prepared as in G and from which CARMIL2 or CARMIL2^QE^ proteins were IP with an anti-CARMIL2 antibody, and subjected to immunoblot analysis with anti-CARMIL2 or CARD11. **(I)** Quantitation of the immunoblot analysis shown in H. Bars represent normalized CARD11-CARMIL2 units. **(J)** CD4^+^ T cells from WT, *Carmil2*^OST^, and *Carmil2*^QE-OST^ mice were left untreated (−) or stimulated with either of the anti-CD3 antibodies 2C11 and 17A2 (+) in the presence (+) or absence (−) of the anti-CD28 antibodies MAB4832 for 2 min prior to isolation of whole-cell lysates. Equivalent amounts of lysates were separated by SDS-PAGE and analyzed by immunoblot with an antibody specific for pTyr. Inducible phosphorylation of SLP76 pTyr128 (pSLP76), LAT pTyr171 (pLAT), and ERK1/2 pThr202/Tyr204 (pERK1/2) was also assessed by immunoblotting with phospho-specific antibodies. In A, B, D, E, G, H, and J, molecular weights in kilodaltons are shown in the left margin. Prior to biochemical analysis, comparable levels of TCRβ, CD3, and CD28 were found at the surface of T cells from WT, *Carmil2*^OST^, *Carmil2*^QE-OST^, OT-I *Carmil2*, OT-I *Carmil2*^QE^, and *Carmil2*^QE^*Cd28*^−/−^mice (Fig. S2 A). Data in A–J are representative of two independent experiments. AP, affinity purification; TL, total lysates; IP, immunoprecipitated; pTyr, phosphotyrosine. Source data are available for this figure: SourceData F2.

Considering that the glutamine residue corresponding to the human *CARMIL2*^QE^ mutation and its flanking residues are conserved in mouse (Zwolak et al., 2013), we determined next whether resting mouse T cells homozygous for a *Carmil*2^QE^ mutation orthologous to the human *CARMIL2*^QE^ mutation contained preformed CARMIL2-CARD11 complexes. Accordingly, two lines of gene-targeted mice expressing either CARMIL2 or CARMIL2^QE^ molecules tagged with an OST sequence were developed (Fig. S1 and Materials and methods), and their naive T cells expressed the tagged proteins at levels comparable to WT CARMIL2 proteins (Fig. 2 A). CD4^+^ T cells purified from WT, *Carmil2*^OST^, and *Carmil2*^QE-OST^ mice were left unstimulated or stimulated with anti-CD3 plus anti-CD28 for 2 and 5 min, and the CARMIL2^OST^ and CARMIL2^QE-OST^ molecules were subjected to affinity purification and analyzed by immunoblots (Fig. 2, B and C). The association between CARMIL2^OST^ molecules and CARD11 required TCR-CD28 stimulation, whereas CARMIL2^QE-OST^ molecules associated with CARD11 prior to TCR-CD28 stimulation. Preformed CARMIL2^QE^-CARD11 complexes were also specifically present in unstimulated CD8^+^ T cells expressing the *Carmil2*^QE^ mutation (Fig. 2, D–F). As observed in Jurkat T cells (Fig. 1, D and E), the levels of preformed CARMIL2^QE-OST^-CARD11 complexes present in unstimulated CD4^+^ and CD8^+^ T cells from *Carmil2*^QE-OST^ mice further increased after pervanadate treatment, a surrogate for TCR-CD28 stimulation (Roncagalli et al., 2014), or TCR-CD28 engagement (Fig. 2, B and E).

To determine whether the assembly of the preformed CARMIL2^QE^-CARD11 complexes found in *Carmil2*^QE^ mouse T cells required the expression of CD28 molecules, *Carmil2*^QE^ mice lacking CD28 molecules (*Carmil2*^QE^*Cd28*^−/−^ mice) were generated and their CD4^+^ T cells were purified and left unstimulated or stimulated with pervanadate. Prior to stimulation, CARMIL2^QE^-CARD11 complexes were also found in *Carmil2*^QE^*Cd28*^−/−^ CD4^+^ T cells at levels twofold lower than those present in unstimulated *Carmil2*^QE^ CD4^+^ T cells, and they further increased after pervanadate stimulation (Fig. 2, H and I). Therefore, CD28 expression is dispensable for the generation of preformed CARMIL2^QE^-CARD11 complexes in unstimulated mouse T cells and for their subsequent increase following TCR activation.

### CARMIL2^QE^ expression does not affect TCR signals

Comparison of *Carmil2*^OST^ and *Carmil2*^QE-OST^ mouse T cells after cross-linkage of the TCR-CD3 complex alone showed that the expression of CARMIL2^QE^ had no detectable effect on the global pattern of TCR-inducible tyrosine-phosphorylated species (Fig. 2 J). Moreover, cross-linking of the TCR-CD3 complex of *Carmil2*^OST^ and *Carmil2*^QE-OST^ T cells induced similar levels of phosphorylation of the ERK serine/threonine protein kinase and of the LAT and SLP76 (also known as LCP2) adaptors (Fig. 2 K), three hallmarks of TCR signaling pathway activity. Therefore, the expression of CARMIL2^QE^ molecules modified neither the pattern of global TCR-inducible tyrosine phosphorylation nor TCR-inducible phosphorylation of LAT, SLP76, and ERK, demonstrating that the *Carmil2*^QE^ mutation does not affect TCR signals.

### T_reg_ and invariant NKT cell development differ in their requirement for CARMIL2-dependent signals

**Figure 3.**
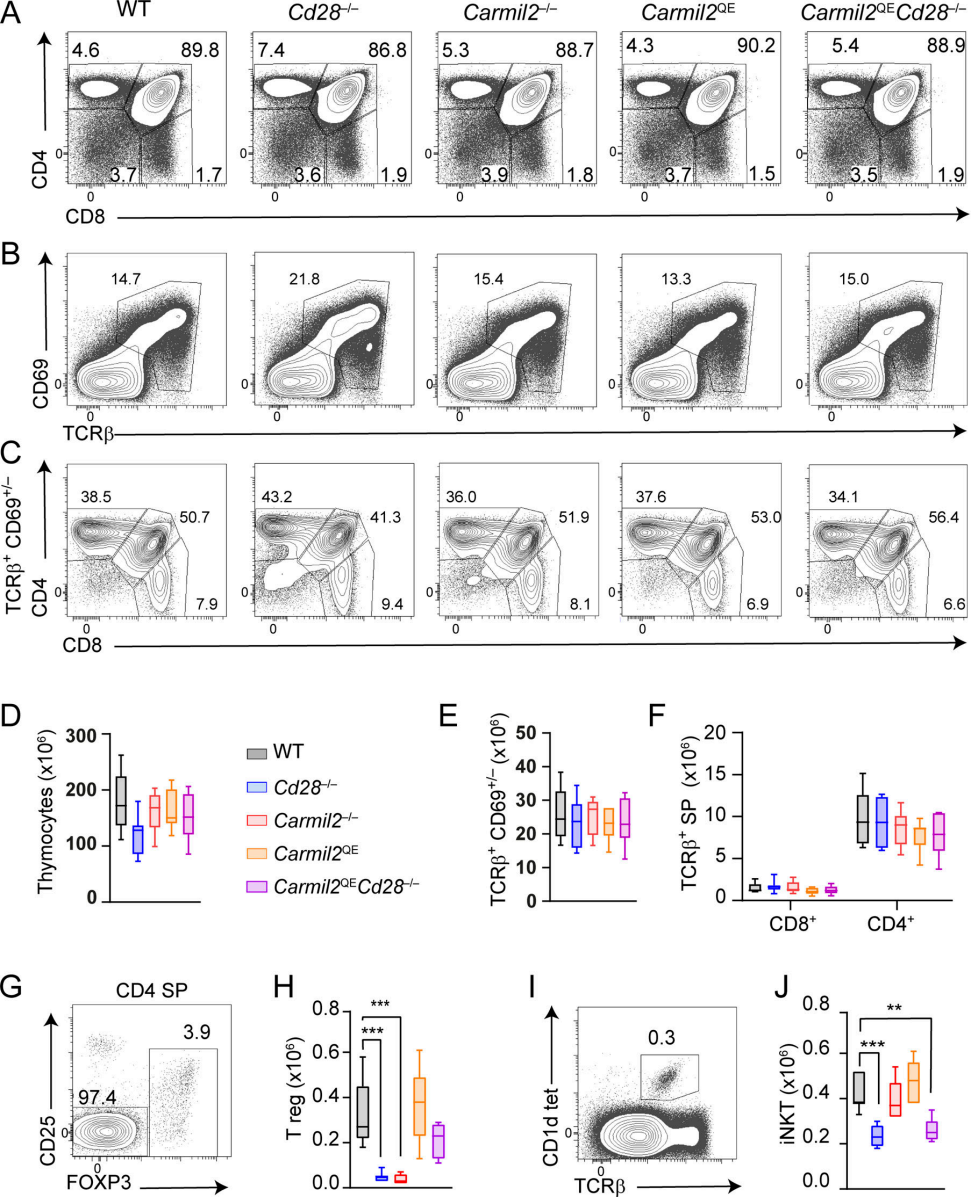
**Effect of the *Carmil2*** ^**QE**^ **mutation on thymic development including T**_**reg**_ **cells and iNKT cells. (A)** WT, *Cd28*^−/−^, *Carmil2*^−/−^, *Carmil2*^QE^, and *Carmil2*^QE^*Cd28*^−/−^ thymi were analyzed by flow cytometry for the expression of CD4 and CD8. Numbers indicate the percentage of CD4^−^CD8^−^ double-negative, CD4^+^CD8^+^ double-positive, and CD4^+^ and CD8^+^ SP cells. **(B)** Analysis of lineage^−^ (CD25^−^, MHCII^−^, CD11b^−^, CD161^−^) thymocytes from the specified thymi using TCRβ-CD69 dot plots. They permit to identify TCRβ^+^CD69^+^ cells that went through TCR-mediated positive and negative selection (Ashby and Hogquist, 2024). Note that TCRβ^hi^CD69^−^ cells were also included in the specified TCRβ^+^CD69^−/+^ gate since they correspond to the most mature SP cells (Hogquist et al., 2015). **(C)** Analysis of CD4 and CD8 expression on gated TCRβ^+^CD69^+/−^ cells showed that they include both DP and SP cells and permit to define CD4^+^ and CD8^+^ mature SP cells. **(D)** Numbers of total cells in thymi isolated from mice of the specified genotypes (see key). **(E)** Numbers of total TCRβ^+^CD69^+/−^ cells in thymi isolated from mice of the specified genotypes (see key in D). **(F)** Numbers of CD8^+^ and CD4^+^ TCRβ^+^CD69^+/−^ mature T cells in thymi isolated from mice of the specified genotypes (see key in D). **(G)** Total CD4^+^ SP cells from WT thymus were analyzed by flow cytometry using FOXP3 and CD25 expression and the percentage of FOXP3^+^CD25^+^ T_reg_ cells among total CD4^+^ SP cells defined using the outlined areas. **(H)** Numbers of FOXP3^+^ T_reg_ cells found in thymi isolated from mice of the specified genotypes (gating strategy as in G). The difference in FOXP3^+^ T_reg_ cell numbers between *Carmil2*^QE^ and *Carmil2*^QE^*Cd28*^−/−^ thymi is not significant. **(I)** WT thymic iNKT cells were analyzed by flow cytometry using TCRβ expression and binding of α-galactosylceramide–complexed CD1d tetramers (CD11d tet), and their percentage among total thymocytes defined using the outlined area. **(J)** Numbers of iNKT cells gated as in I in thymi isolated from mice of the specified genotypes. Data in A–J were pooled from four experiments with a total of eight mice per group. The data in D–F, H, and J are shown as box plots with the median, boxed interquartile, and whiskers. Data were analyzed by two-way ANOVA applying the Holm–Sidàk multiple comparison toward the WT group. Only significant values with P ≤ 0.05 are shown. *P < 0.05, **P < 0.01, ***P < 0.001. DP, double positive; SP, single positive.

To explore whether the *Carmil2*^QE^ mutation can substitute for CD28 during T cell development, we generated mice homozygous for the *Carmil2*^QE^ mutation and expressing (*Carmil2*^QE^) or lacking (*Carmil2*^QE^*Cd28*^−/−^) CD28 molecules, and we compared their thymi with those of age-matched WT-, *Cd28*^−/−^-, and CARMIL2-deficient (*Carmil2*^−/−^) mice. *Carmil2*^QE^ and *Carmil2*^QE^*Cd28*^−/−^ thymi had normal cellularities and frequencies of CD4^–^CD8^–^ double-negative, CD4^+^CD8^+^ double-positive, and CD4^+^ and CD8^+^ single-positive cells (Fig. 3, A and D). It resulted in percentages (Fig. 3, B and C) and numbers (Fig. 3, E and F) of CD8^+^ and CD4^+^ TCRβ^+^CD69^+/−^ mature T cells comparable to those of WT thymi. Considering that thymic T_reg_ cell development requires both TCR- and CD28-CARMIL2-CARD11-mediated signals (Carter and Pomerantz, 2022; Liang et al., 2013; Roncagalli et al., 2016; Tai et al., 2005; Vang et al., 2010), we analyzed whether the preformed CARMIL2^QE^-CARD11 complexes found in naive T cells from *Carmil2*^QE^*Cd28*^−/−^ mice were capable of replacing the need for CD28 engagement during T_reg_ cell development. As expected, thymi from *Cd28*^−/−^ and *Carmil2*^−/−^ mice had approximately eightfold lower numbers of T_reg_ cells as compared to WT thymi (Fig. 3, G and H). In contrast, the numbers of T_reg_ cells in *Carmil2*^QE^ and *Carmil2*^QE^*Cd28*^−/−^ thymi were not significantly different from those of WT thymi, demonstrating that *Carmil2*^QE^ molecules can substitute for CD28 during thymic T_reg_ cell development. The development of thymic invariant natural killer T (iNKT) cells requires TCR engagement and CD28 signals that are delivered independently of CARD11 (Medoff et al., 2009; Williams et al., 2008). Consistent with these results, the lack of CARMIL2 had no deleterious effect on iNKT cell development and the expression of CARMIL2^QE^ molecules in *Carmil2*^QE^*Cd28*^−/−^ thymi failed to restore iNKT cell development (Fig. 3, I and J). Therefore, both thymic T_reg_ and iNKT cells develop in a CD28-dependent manner but differed in their requirement for CARMIL2-CARD11 signals.

Considering that CD28 engagement activates multiple signaling branches (see Discussion), our analysis of T_reg_ and iNKT cell development suggested that whenever the CD28 signals required for a given developmental or functional outcome can be replaced by those triggered by the ready-made CARMIL2^QE^-CARD11 complexes found in *Carmil2*^QE^*Cd28*^−/−^ mice, it can be inferred that the considered CD28-mediated outcome is physiologically driven by the sole CARMIL2-CARD11 signaling branch. Accordingly, we compared next the phenotype of WT, *Cd28*^−/−^, *Carmil2*^−/−^, *Carmil2*^QE^, and *Carmil2*^QE^*Cd28*^−/−^ mice for each of the developmental and functional traits known to require CD28 costimulation. As described below, it allowed to disentangle such traits into those for which CARMIL2-CARD11-driven CD28 signals are (1) necessary and sufficient, (2) necessary but not sufficient, or (3) dispensable (Table S1), providing a unique opportunity to determine the relative importance of CARMIL2-CARD11-driven signals among those triggered by CD28 under physiological in vivo conditions.

### Peripheral T cells in *Carmil2*^QE^*Cd28*^−/−^ mice

**Figure 4.**
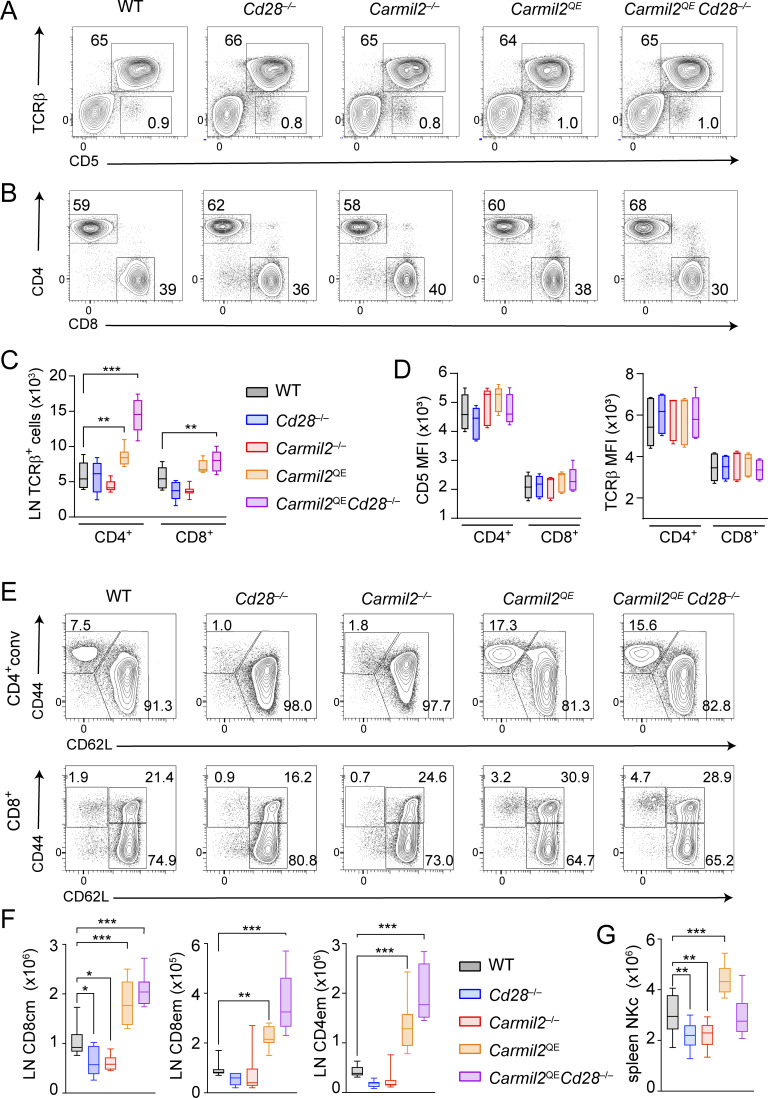
**Effect of the *Carmil2*** ^**QE**^ **mutation on peripheral T cell homeostasis. (A)** Total T cells from LN of WT, *Cd28*^−/−^, *Carmil2*^−/−^, *Carmil2*^QE^, and *Carmil2*^QE^*Cd28*^−/−^ mice were analyzed by flow cytometry for the expression of TCRβ and CD5. Numbers indicate the percentage of cells in the specified quadrants. **(B)** TCRβ^+^ cells from LN of the specified mice (gated as in A) were analyzed by flow cytometry for the expression of CD4 and CD8. Numbers indicate the percentage of cells in the CD4^+^CD8^−^ and CD8^+^CD4^−^ quadrants. **(C)** Numbers of TCRβ^+^CD4^+^ and TCRβ^+^CD8^+^ T cells in LN of mice of the specified genotypes (see key). **(D)** MFI of CD5 and TCRβ expression on CD4^+^ T_conv_ and CD8^+^ T cells isolated from the LN of the specified mice (see key in C). **(E)** CD4^+^ T_conv_ (CD4^+^conv) cells and CD8^+^ T cells from LN of WT, *Cd28*^−/−^, *Carmil2*^−/−^, *Carmil2*^QE^, and *Carmil2*^QE^*Cd28*^−/−^ mice were analyzed by flow cytometry for the expression of CD44 and CD62L. It allows to segregate CD4^+^ T cells into naive (CD44^lo^CD62L^hi^) and effector-memory (CD44^hi^CD62L^lo^) cells, and CD8^+^ T cells into naive (CD44^lo^CD62L^hi^) and antigen-experienced CD44^hi^, which comprise central memory (CD62L^hi^) and effector-memory (CD62L^lo^) CD8^+^ T cells. Numbers indicate the percentage of cells in the specified quadrants. **(F)** Numbers of central memory (cm) and of effector-memory (em) CD8^+^ and CD4^+^ T_conv_ cells in the LN of the specified mouse (see key). **(G)** Numbers of CD161^+^ NK cells in the spleen of the specified mouse (see key in F). Data in A–G were pooled from four experiments with a total of eight mice per group. Data in C, E, and F were analyzed by one- or two-way ANOVA applying the Holm–Sidàk multiple comparison toward the WT group. Only significant values with P ≤ 0.05 are shown in black, and values comparing *Carmil2*^QE^ and *Carmil2*^QE^*Cd28*^−/−^ mice are shown in red. *P < 0.05, **P < 0.01, ***P < 0.001. MFI, mean fluorescence intensity.

*Carmil2* ^QE^ and *Carmil2*^QE^*Cd28*^−/−^ lymph nodes (LN) had normal cellularities and frequencies of CD4^+^ conventional T (T_conv_) cells and CD8^+^ T cells that expressed normal levels of TCRβ and CD5 (Fig. 4, A–D). *Carmil2*^QE^ and *Carmil2*^QE^*Cd28*^−/−^ LN primarily differed from WT LN by the presence of up to fourfold increased numbers of effector-memory CD4^+^ T (Fig. 4, E and F). Central and effector-memory CD8^+^ T cell numbers were also two and fourfold increased, respectively, in *Carmil2*^QE^ and *Carmil2*^QE^*Cd28*^−/−^ LN as compared to WT LN (Fig. 4, E and F). Immature mouse NK cells co-express CD28 and CARMIL2 (Roncagalli et al., 2016), and CD28 contributes to their optimal proliferation in response to IL-2 (Hunter et al., 1997; Nandi et al., 1994). The spleen of *Cd28*^−/−^ and *Carmil2*^−/−^ mice contained 1.5-fold reduced numbers of the NK cell as compared to the WT spleen, whereas the *Carmil2*^QE^*Cd28*^−/−^ spleen expressed normal NK cell numbers (Fig. 4 G). Therefore, CARMIL2-CARD11-mediated CD28 signals were necessary and sufficient for proper NK cell homeostasis.

**Figure 5.**
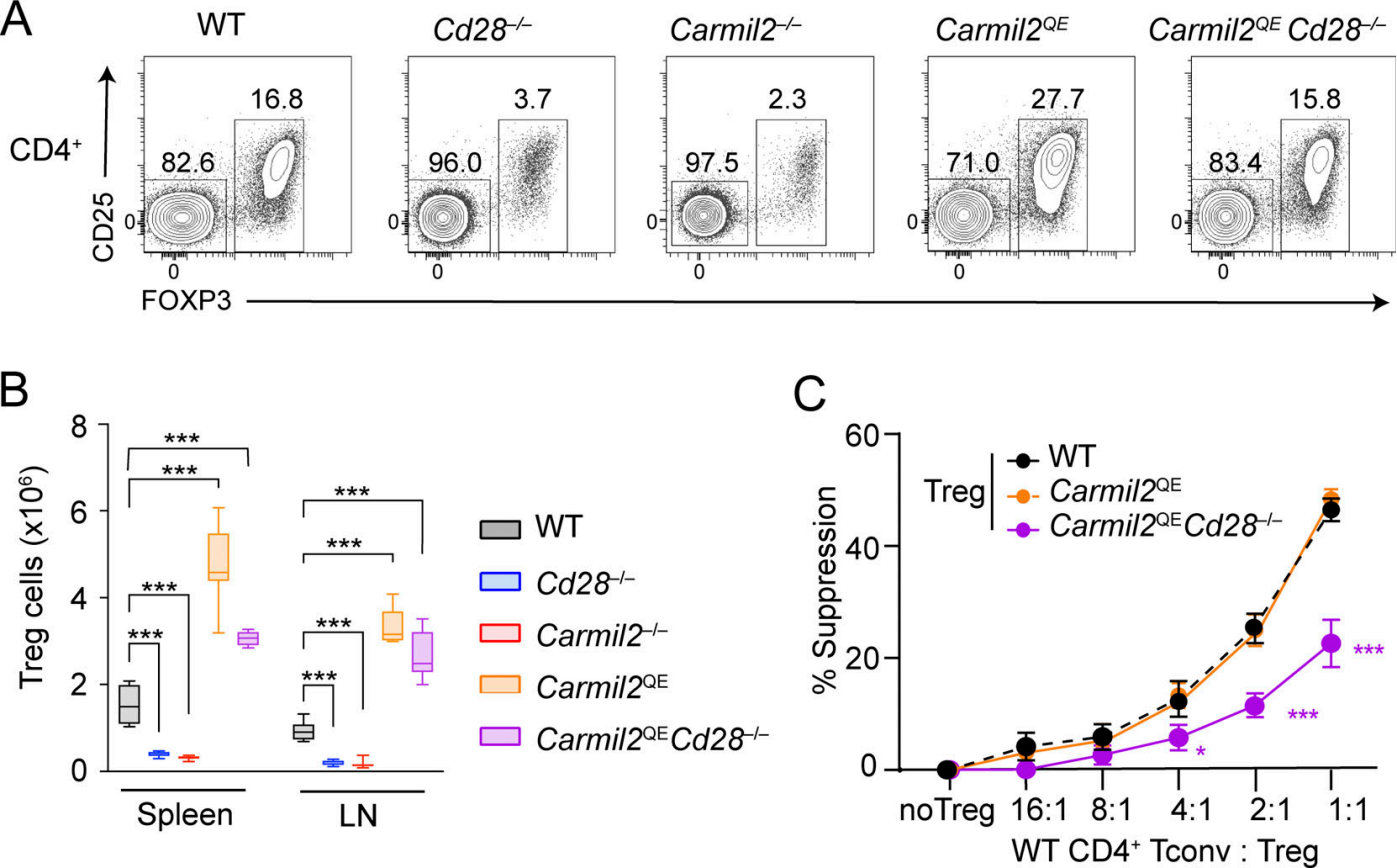
**Effect of the *Carmil2*** ^**QE**^ **mutation on peripheral T**_**reg**_ **cell homeostasis and suppressive function. (A)** Total CD4^+^ T cells from LN of the specified mice were analyzed by flow cytometry for the expression of CD25 and FOXP3. The FOXP3^+^CD25^lo to high^ gate corresponds to T_reg_ cells, and their percentages are shown. **(B)** Numbers of T_reg_ cells in the spleen and LN of the specified mouse (see key). Data were pooled from four experiments with a total of 14 mice per group. Data are shown as box plots with the median, boxed interquartile, and whiskers. **(C)** Sorted T_reg_ cells from WT, *Carmil2*^QE^, and *Carmil2*^QE^*Cd28*^−/−^ spleens were cultured at the indicated ratio with CTV-labeled CD4^+^CD25^−^ T_conv_ cells from WT mice in the presence of anti-CD3-CD28-coated beads, and the percentage of WT CD4^+^CD25^−^ T_conv_ cells that have divided was evaluated after 72 h of culture (see Materials and methods). Data were pooled from four experiments with a total of six mice per group. Percent suppression was calculated using the following formula: $\left(\text{proliferation}\;\text{of}\;T_{\text{conv}}\;\text{cells}\;\text{alone}\;–\text{proliferation}\;\text{of}\;T_{\text{conv}}\;\text{cells}\;\text{treated}\;\text{with}\;T_{\text{reg}}\;\text{cells}\right)/\left(\text{proliferation}\;\text{of}\;T_{\text{conv}}\;\text{cells}\;\text{alone}\right)\times 100$. Mean value ± SEM are represented. Data in B and C were analyzed by one- or two-way ANOVA applying the Holm–Sidàk multiple comparison toward the WT group. Only significant values with P ≤ 0.05 are shown in black. *P < 0.05, ***P < 0.001.

Consistent with the diminished numbers of T_reg_ cells developing in *Cd28*^−/−^ and *Carmil2*^−/−^ thymi, the spleen and LN of *Cd28*^−/−^ and *Carmil2*^−/−^ mice contained fourfold reduced numbers of T_reg_ cells as compared to their WT counterparts (Fig. 5, A and B). Considering that the selective ablation of CD28 on peripheral T_reg_ cells diminished both their survival and optimal maturation into effector T_reg_ cells (Zhang et al., 2015), we analyzed the T_reg_ cells that develop normally in *Carmil2*^QE^*Cd28*^−/−^ thymi and seed the LN and spleen and found that their numbers were threefold increased as compared to their WT counterpart (Fig. 5, A and B). They had, however, a twofold reduced suppressive activity as compared to those of WT and *Carmil2*^QE^ mice (Fig. 5 C). Therefore, CARMIL2-CARD11-mediated CD28 signals were necessary and sufficient for the survival of peripheral T_reg_ cell but insufficient to endow them with optimal suppressive activity.

### CARMIL2^QE^ expression enhances suboptimal TCR signals in the absence of CD28

**Figure 6.**
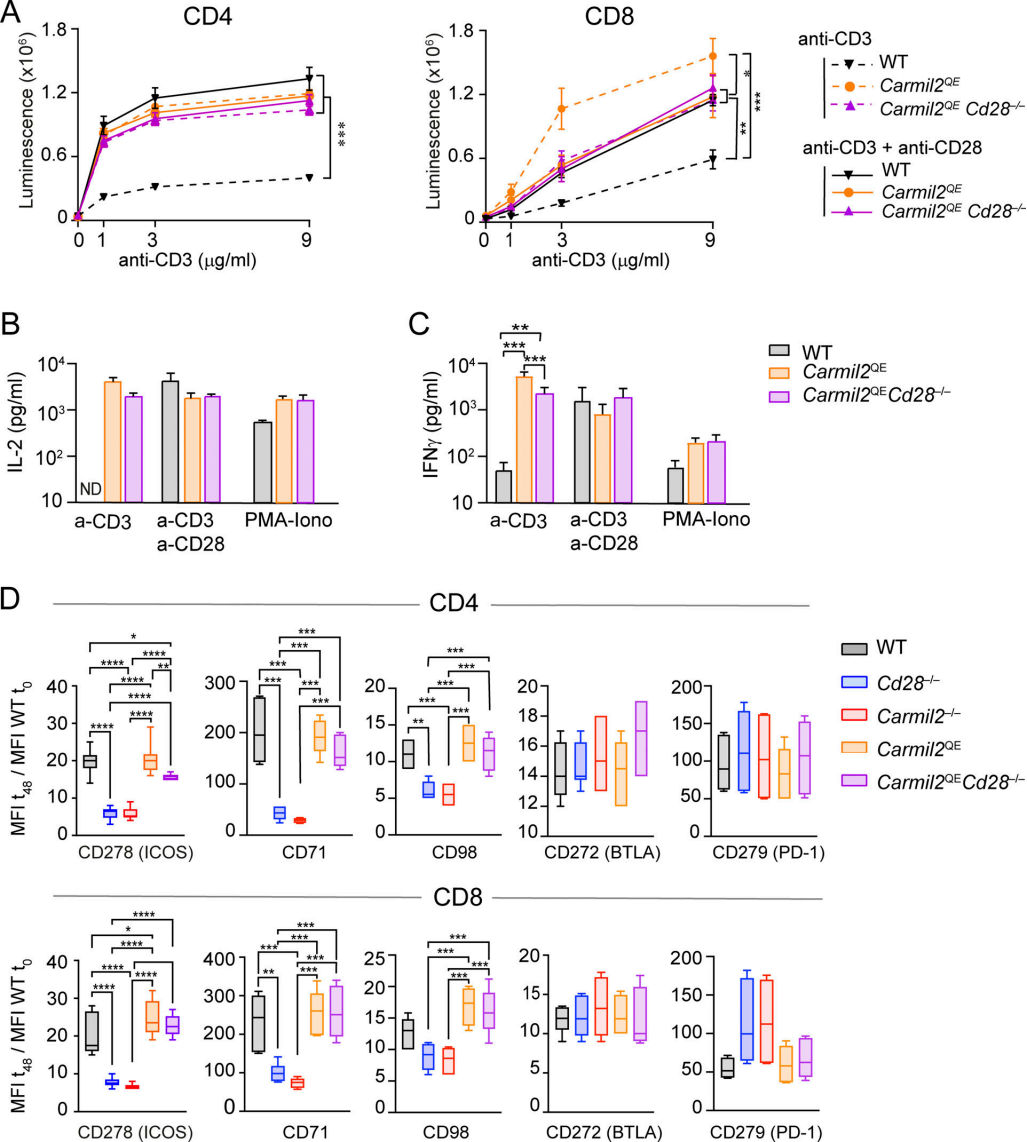
**CARMIL2-CARD11-dependent signals maximize TCR-induced proliferation, cytokine production, and expression of activation markers. (A)** Naive CD4^+^ and CD8^+^ T cells purified from the spleen and LN of WT, *Carmil2*^QE^, and *Carmil2*^QE^*Cd28*^−/−^mice were activated in vitro with the specified concentrations of plate-bound anti-CD3 in the presence or absence of a fixed concentration (1 μg/ml) of soluble anti-CD28. CD4^+^ and CD8^+^ T cell proliferation was measured by luminescence after 48 h. Stimulation of *Carmil2*^QE^ CD8^+^ T cells with anti-CD3 resulted in twofold increased proliferation as compared to anti-CD3–stimulated *Carmil2*^QE^*Cd28*^−/−^ CD8^+^ T cells, the reason for which remains to be elucidated. Data were analyzed by one-way ANOVA applying the Holm–Sidàk multiple comparison toward the specified groups. *P < 0.05, **P < 0.01, ***P < 0.001. **(B)** Naive CD4^+^ T cells from the specified mice (see key in C) were activated as in A with 3 μg/ml of anti-CD3 in the presence or absence of 1 μg/ml anti-CD28 and the content of IL-2 present in the supernatant of 40-h-long coculture assessed. Also shown is the IL-2 produced upon stimulation with PMA and ionomycin. **(C)** Naive CD8^+^ T cells from the specified mice were activated as in A with 3 μg/ml of anti-CD3 in the presence or absence of 1 μg/ml anti-CD28 and the content of IFN-γ present in the supernatant of 40-h-long coculture assessed (see key). Data in A–C were pooled from two experiments out of four, each with a total of four mice per group. Mean value ± SEM are represented; ND indicates nondetectable IL-2 level. Data in B and C were analyzed by one-way ANOVA applying the Holm–Sidàk multiple comparison toward the specified groups. Only significant values with P ≤ 0.05 are shown. **P < 0.01, ***P < 0.001. **(D)** Naive CD4^+^ and CD8^+^ T cells purified from the spleen and LN of the specified mice (see key) were activated in vitro using anti-CD3 (3 μg/ml) plus anti-CD28 (1 μg/ml) cross-linkage, and the levels of CD278 (ICOS), CD71, CD91, CD272 (BTLA), and CD279 (PD-1) were determined by flow cytometry. The ratio of the MFI at t_48h_ to the MFI at t_0_h is shown for each of the analyzed molecules. Data in D are shown as box plots with the median, boxed interquartile, and whiskers, and pooled from two experiments, each with a total of six mice per group. Data in D were analyzed by two-way ANOVA applying the Holm–Sidàk multiple comparison toward the specified groups. Only significant values with P ≤ 0.05 are shown. *P < 0.05, **P < 0.01, ***P < 0.001, ****P < 0.0001. Histograms corresponding to the levels of CD278 (ICOS), CD71, CD91, CD272 (BTLA), and CD279 (PD-1) on CD4^+^ and CD8^+^ T cells from WT, *Carmil2*^OST^, and *Carmil2*^QE-OST^ mice prior to and after 48 h of activation are shown in Fig. S2 B. MFI, mean fluorescence intensity.

Stimulation of WT, *Cd28*^−/−^, and *Carmil2*^−/−^ naive T cells with suboptimal concentrations of anti-CD3 in the presence or absence of a fixed concentration of anti-CD28 showed that the lack of CARMIL2 molecules prevented CD28 from enhancing suboptimal TCR signals as the lack of CD28 did (Liang et al., 2013). Using the same assay, naive CD4^+^ and CD8^+^ T cells isolated from *Carmil2*^QE^*Cd28*^−/−^ mice and stimulated with anti-CD3 alone proliferated and produced IL-2 and interferon-γ (IFN-γ) at levels comparable to WT T cells stimulated with both anti-CD3 and anti-CD28 (Fig. 6, A–C). Therefore, CARMIL2-CARD11-mediated CD28 signals were necessary and sufficient to enhance the suboptimal signals resulting from low TCR occupancy and it led to normal levels of T cell proliferation and cytokine production.

### CARMIL2^QE^ expression enhances activation marker induction on naive T cells in the absence of CD28

**Figure S2.**
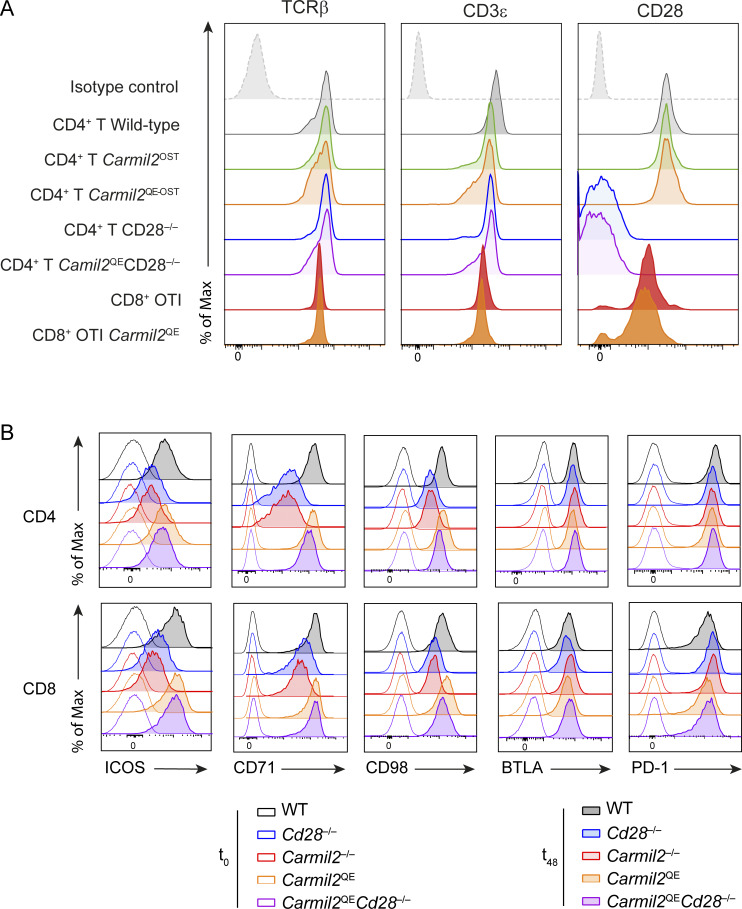
**Comparative expression of cell surface markers on CD4** ^**+**^ **and CD8** ^**+**^ **T cells of WT, *Carmil2*** ^**OST**^ **, *Carmil2*** ^**QE-OST**^ **, OT-I *Carmil2*, OT-I *Carmil2*** ^**QE**^ **, and *Carmil2*** ^**QE**^ ***Cd28*** ^**−/−**^ **mice. (A)** Related to Fig. 2. Naive CD4^+^ and CD8^+^ T cells purified from the spleen and LN of WT, *Carmil2*^OST^, *Carmil2*^QE-OST^, OT-I *Carmil2*, OT-I *Carmil2*^QE^, and *Carmil2*^QE^*Cd28*^−/−^ mice were analyzed by flow cytometry for the levels of TCR, CD3, and CD28 prior to biochemical analysis (shaded curves). Dashed line curves correspond to isotype-matched control antibodies (negative controls), and data are representative of two independent experiments. **(B)** Related to Fig. 6. Naive CD4^+^ and CD8^+^ T cells purified from the spleen and LN of WT, *Cd28*^−/−^, *Carmil2*^−/−^, *Carmil2*^QE^, and *Carmil2*^QE^*Cd28*^–/–^mice (see key) were activated in vitro using anti-CD3 plus anti-CD28 cross-linkage, and the levels of ICOS (CD278), CD71, CD98, BTLA (CD272), and PD-1 (CD279) were measured by flow cytometry prior to activation (t_0_, solid line curves) or after 48 h of activation (t_48_, shaded curves). Data are representative of two experiments, each with a total of six mice per genotype.

Upon TCR-CD28 engagement, CD28 signals enhance the expression of several receptors at the surface of naive T cells including the ICOS costimulatory receptor (also known as CD278) and transporters that carry iron (CD71) or amino acids (CD98) into T cells. Likewise, coinhibitory molecules such as BTLA (CD272) and PD-1 (CD279) are also transiently induced to prevent excessive T cell responses. The respective expression levels of such molecules were then measured on naive CD4^+^ and CD8^+^ T cells isolated from WT, *Cd28*^−/−^, *Carmil2*^−/−^, *Carmil2*^QE^, and *Carmil2*^QE^*Cd28*^−/−^ mice prior to and following anti-CD3 plus anti-CD28 cross-linkage for 48 h (Fig. 6 D). CD4^+^ and CD8^+^ T cells from *Cd28*^−/−^ and *Carmil2*^−/−^ mice expressed reduced levels of ICOS as compared to WT CD4^+^ and CD8^+^ T cells (Fig. 6 E and Fig. S2 B). In contrast, CD4^+^ and CD8^+^ T cells from *Carmil2*^QE^*Cd28*^−/−^ mice expressed ICOS at levels comparable to WT T cells. A similar pattern of responses was observed for CD71 and CD98, whereas induction of BTLA and PD-1 was found less dependent on CD28 signals. Therefore, CARMIL2-CARD11-mediated CD28 signals were necessary and sufficient to trigger the expression of normal levels of ICOS, CD71, and CD98 at the surface of naive *Carmil2*^QE^*Cd28*^−/−^ T cells following TCR stimulation.

### CARMIL2^QE^ expression renders OT-I T cell activation independent of CD28 engagement

**Figure S3.**
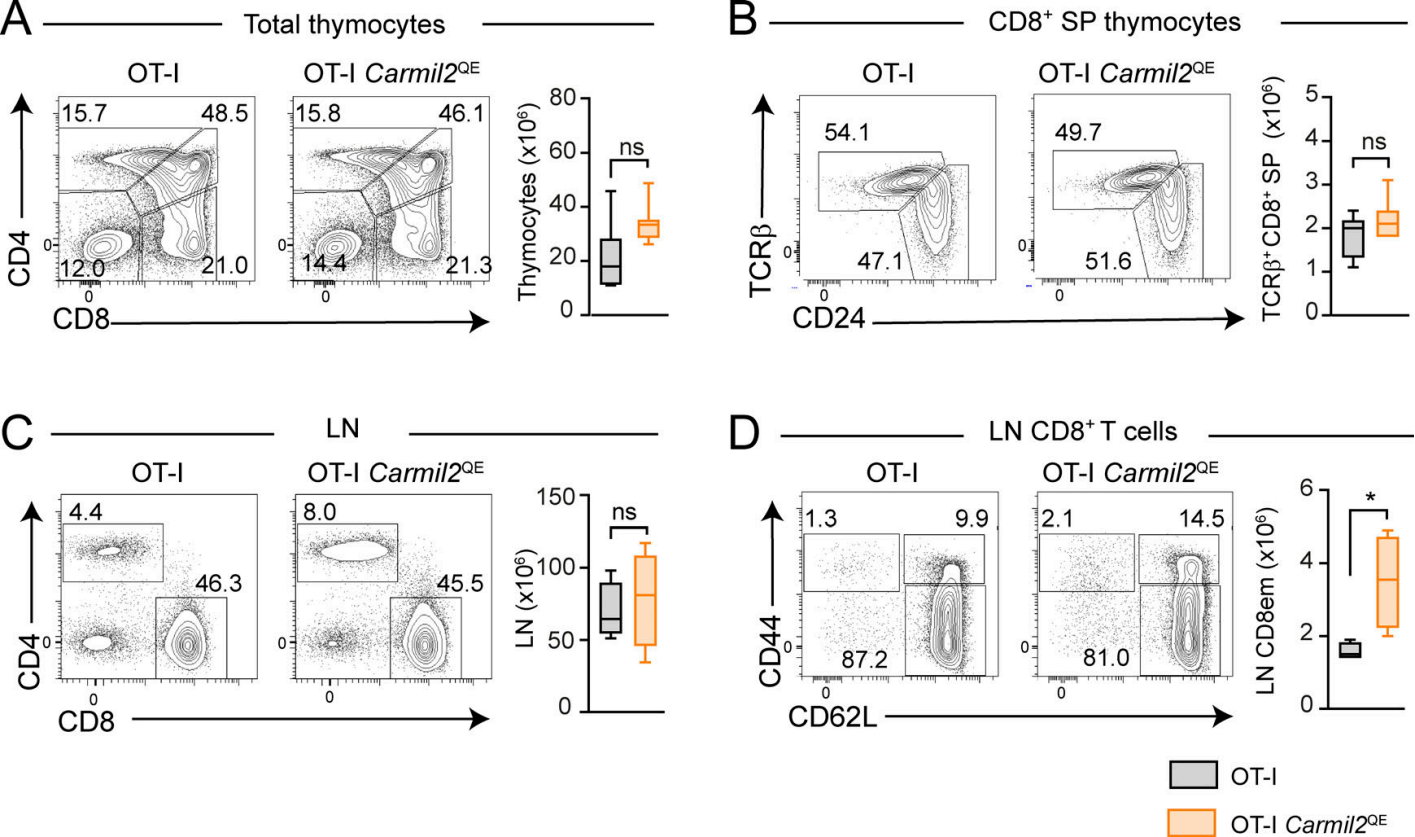
**OT-I T cells develop normally in the presence of CARMIL2** ^**QE**^ **molecules. (A)** Total cells from OT-I and OT-I *Carmil2*^QE^ thymi were analyzed by flow cytometry for the expression of CD4 and CD8. Numbers indicate the percentage of CD4^−^CD8^−^ double-negative, CD4^+^CD8^+^ double-positive, and CD4^+^ and CD8^+^ SP cells. Also shown on the right is the cellularity of OT-I and OT-1 *Carmil2*^QE^ thymi. **(B)** Total cells from OT-I and OT-1 *Carmil2*^QE^ thymi were analyzed for the expression of TCRβ and CD24. It permits to distinguish mature CD8^+^ T cells (TCRβ^+^ CD24) and immature SP CD8^+^ T cells (TCRβ^−^CD24^+^), the percentages of which are indicated by the number adjacent to outlined areas. Also shown on the right is the quantification of the numbers of TCRβ^+^ CD24^–^ mature CD8^+^ T cells found in OT-I and OT-1 *Carmil2*^QE^ thymi. **(C)** T cells from OT-I and OT-1 *Carmil2*^QE^ LN were analyzed by flow cytometry for the presence of CD4^+^ and CD8^+^ T cells, the percentages of which are indicated by the number adjacent to outlined areas. Also shown on the right is the quantification of the numbers of T cells in OT-I and OT-1 *Carmil2*^QE^ LN. **(D)** Gated CD8^+^ T cells from LN of OT-I and OT-1 *Carmil2*^QE^ mice (see C) were analyzed using CD44 and CD62L expression. The percentages of naive (CD44^lo^CD62L^hi^), effector-memory (CD44^hi^ CD62L^lo^), and central memory (CD44^hi^ CD62L^hi^) CD8^+^ T cells are indicated by the number adjacent to outlined areas. Also shown on the right is the quantification of the numbers of CD8^+^ effector-memory T cells in OT-I and OT-1 *Carmil2*^QE^ LN. Data in A–D were pooled from three independent experiments with a total of six mice per group. Data quantification is shown as box plots with the median, boxed interquartile, and whiskers. Data were analyzed by two-way ANOVA applying the Holm–Sidàk multiple comparison toward the OT-I group. *P < 0.05, ns, nonsignificant. SP, single positive.

**Figure 7.**
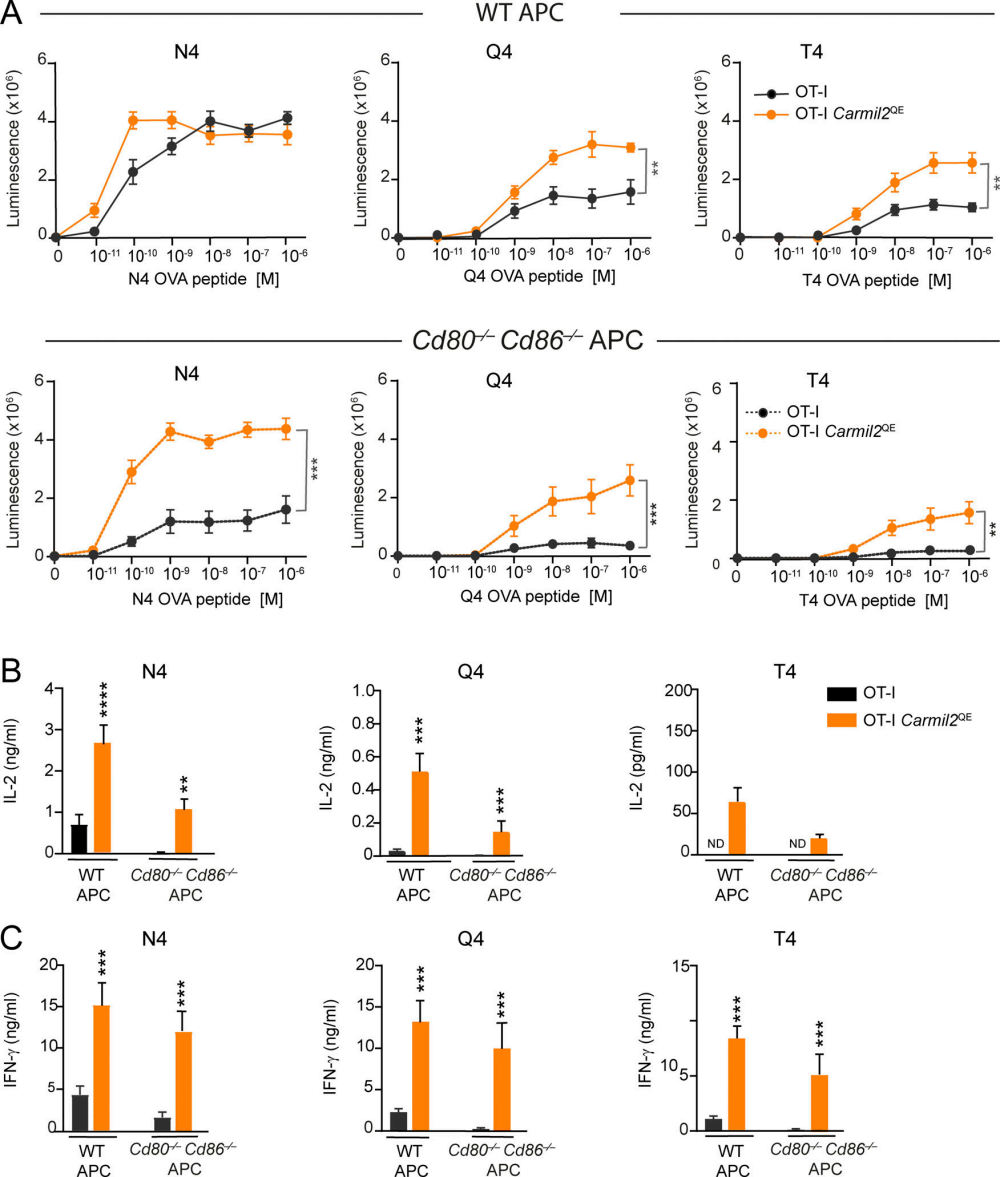
**CARMIL2-CARD11-dependent signals replace CD28 engagement during antigen-induced proliferation and cytokine production. (A)** Naive CD8^+^ T cells purified from OT-I and OT-I *Carmil2*^QE^ spleens were stimulated with APC (corresponding to dendritic cells and B cells) isolated from the spleen of T cell–deficient *Cd3*e^Δ5/Δ5^ mice expressing (WT APC) or lacking CD80 and CD86 (*Cd80*^−/−^*Cd86*^−/−^ APC; see key). APC were pulsed for 2 h with a graded concentration of OVA peptides corresponding to agonist (N4) or weak agonist (Q4 and T4) OVA peptides and used to stimulate OT-I and OT-I *Carmil2*^QE^ T cells. T cell proliferation was measured by luminescence after 48 h. **(B)** Naive OT-I and OT-I *Carmil2*^QE^ T cells were stimulated as in A with the N4, Q4, and T4 peptides (10^−6^ M) and the content of IL-2 present in the supernatant of 40-h-long coculture assessed (see key). **(C)** Naive OT-I and OT-I *Carmil2*^QE^ T cells were stimulated as in A with N4, Q4, and T4 peptides (10^−6^ M) and the content of IFN-γ present in the supernatant of 40-h-long coculture assessed (see key in B). Data were pooled from four experiments, each with a total of five mice per group. Mean value ± SEM are represented; ND indicates nondetectable IL-2 and IFN-γ levels. Data were analyzed by one-way ANOVA applying the Holm–Sidàk multiple comparison toward the OT-I group. Only significant values with P ≤ 0.05 are shown. **P < 0.01, ***P < 0.001, ****P < 0.0001.

To assess whether CARMIL2^QE^ molecules can substitute for CD28 engagement during T cell responses induced by antigen-laden APC, mice expressing the OT-I TCR specific for the N4 ovalbumin peptide (OVA257-264; Barnden et al., 1998) were backcrossed onto mice expressing CARMIL2^QE^ molecules (OT-I *Carmil*2^QE^ mice). OT-I *Carmil*2^QE^ T cells developed similar to OT-I cells (Fig. S3). APC isolated from the spleen of CD3ε-deficient mice and expressing (*Cd3*ε^Δ5/Δ5^ mice) or lacking (*Cd3*ε^Δ5/Δ5^*Cd80*^−/−^*Cd86*^−/−^ mice) CD80 and CD86 molecules were irradiated and pulsed with agonist (N4) or partial agonist (Q4 and T4) OVA peptides and cultured for 48 h with naive CD8^+^ T cells isolated from OT-I and OT-I *Carmil*2^QE^ mice. The ensuing T cell proliferation and IL-2 and IFN-γ cytokine production were then measured. The expression of CD80-CD86 on APC was essential to maximize WT OT-I T cell proliferation at all tested N4 peptide concentrations (Fig. 7 A). In marked contrast, OT-I *Carmil*2^QE^ T cells stimulated with N4-pulsed APC lacking CD80-CD86 expression proliferated at levels comparable—or slightly higher at low N4 concentrations—to those observed for WT OT-I T cells stimulated with N4-pulsed CD80-CD86-expressing APC. The ability of the *Carmil*2^QE^ mutation to replace CD28 costimulation was even more blatant for IL-2 and IFN-γ production in response to the weak Q4 and T4 agonists (Fig. 7, B and C). Therefore, CARMIL2-CARD11-mediated CD28 signals were necessary and sufficient to maximize the proliferation and cytokine production of naive OT-I *Carmil*2^QE^ T cells in response to CD80-CD86-deficient APC laden with agonist and weak agonist OVA peptides. Importantly, the *Carmil2*^QE^ mutation induced no OT-I proliferation or cytokine production in the absence of anti-CD3 cross-linkage (Fig. 6) or of cognate antigen (Fig. 7), demonstrating that its functional manifestations remained dependent on TCR engagement.

### CARMIL2^QE^ expression replaces CD28 during antitumor T cell responses

**Figure 8.**
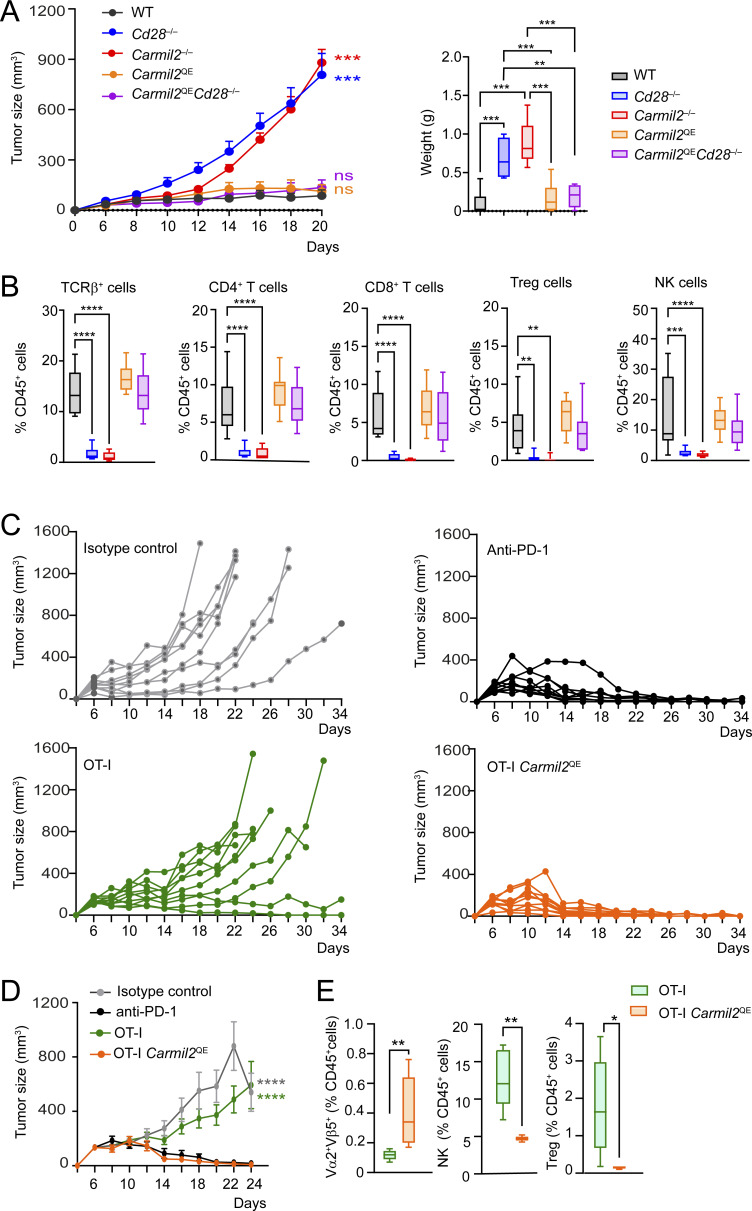
**Expression of CARMIL2** ^**QE**^ **molecules substitutes for CD28 engagement during responses to solid tumors. (A)** Cohorts of 10-wk-old, WT, *Cd28*^−/−^, *Carmil2*^−/−^, *Carmil2*^QE^, and *Carmil2*^QE^*Cd28*^−/−^ C57BL/6 mice were injected subcutaneously into the flank with 1 × 10^5^ cells of the BRAF^V600E^*Ptgs*^−/−^ syngeneic melanoma tumor and monitored for tumor growth using tumor size and weight. The mean and SEM are shown for the tumor size values, and the weight panel corresponds to box plots with the median, boxed interquartile, and whiskers. Data were pooled from three independent experiments with a total of 12–14 mice per group. **(B)** Immune cell infiltrate analysis of BRAF^V600E^*Ptgs1/Ptgs2*^*−/−*^ tumors 11 days after implantation in WT, *Cd28*^−/−^, *Carmil2*^−/−^, *Carmil2*^QE^, and *Carmil2*^QE^*Cd28*^–/–^mice. The percentages of intratumoral TCRβ^+^ cells, CD4^+^ T cells, CD8^+^ T cells, T_reg_ cells, and NK cells among CD45^+^ cells are shown (see key in A). Box plots with the median, boxed interquartile, and whiskers are shown, and data were pooled from three independent experiments, each with a total of nine mice per group. **(C)** Tumor growth analysis in WT mice injected subcutaneously with MC38-OVA carcinoma cells and treated with isotype control antibody, anti-PD-1 antibody, OT-I T cells, or *Carmil2*^Q538E^ OT-I T cells 6 days after tumor inoculation. The lines indicate tumor volume over time in individual mice up to the time they had to be euthanized. Tumor growth was monitored three times a week. Data are representative of two independent experiments each involving 10 mice per condition. **(D)** Results in C were expressed as mean tumor volume (mm^3^ ± SEM) and P values shown for day 24. **(E)** Analysis of immune cell infiltrates of MC38-OVA tumors 11 days after implantation in mice that have been infused with OT-I T cells and *Carmil2*^QE^ OT-I T cells. The percentages of Vα2^+^Vβ5^+^ OT-I T cells, Vα2^+^Vβ5^+^*Carmil2*^QE^ OT-I T cells, NK cells, and T_reg_ cells among intratumoral CD45^+^ cells are shown. Data in A, B, D, and E were analyzed by one-way or two-way ANOVA applying the Holm–Sidàk multiple comparison toward the specified groups. Only significant values with P ≤ 0.05 are shown. *P < 0.05, **P < 0.01, ***P < 0.001, ****P < 0.0001.

Considering that CD28 costimulation plays an essential role in the establishment of antitumor T cell responses (Agarwal et al., 2023; Duraiswamy et al., 2021; Kamphorst et al., 2017; Magen et al., 2023), we determined next whether the expression of CARMIL2^QE^ proteins can replace CD28 engagement during responses to solid tumors. Cohorts of WT, *Cd28*^−/−^, *Carmil2*^−/−^, *Carmil2*^QE^, and *Carmil2*^QE^*Cd28*^−/−^ mice were injected subcutaneously with the syngeneic mouse melanoma tumor BRAF^V600E^*Ptgs*^−/−^ and monitored for tumor growth (Fig. 8 A). The BRAF^V600E^*Ptgs*^−/−^ tumor is immunogenic in WT mice (Zelenay et al., 2015), and in turn, all the mice belonging to the WT cohort rejected the tumor cells. Analysis of *Cd28*^−/−^ and *Carmil2*^−/−^ mouse cohorts showed that both CD28 and CARMIL2 molecules were essential for antitumor rejection since their absence permitted BRAF^V600E^*Ptgs*^−/−^ tumor growth, whereas *Carmil2*^QE^ mice rejected the BRAF^V600E^*Ptgs*^−/−^ tumor as efficiently as WT mice. Importantly, the expression of CARMIL2^QE^ molecules in *Carmil2*^QE^*Cd28*^−/−^ mice was capable of substituting for CD28 engagement and led to complete tumor rejection. Comparison of the immune cells infiltrating BRAF^V600E^*Ptgs*^−/−^ tumors of WT, *Cd28*^−/−^, *Carmil2*^−/−^, *Carmil2*^QE^, and *Carmil2*^QE^*Cd28*^−/−^ mice 11 days after inoculation showed that BRAF^V600E^*Ptgs*^−/−^ tumors from *Cd28*^−/−^ and *Carmil2*^−/−^ mice contained reduced percentages of CD4^+^ and CD8^+^ T cells, T_reg_ cells, and NK cells as compared to tumors from WT mice (Fig. 8 B). In contrast, the expression of CARMIL2^QE^ molecules in *Carmil2*^QE^*Cd28*^−/−^ mice resulted in a constellation of tumor-infiltrating lymphocytes, the composition and size of which were similar to those of the tumors implanted on WT mice. Therefore, CARMIL2-CARD11-mediated CD28 signals were necessary and sufficient to eradicate the BRAF^V600E^*Ptgs*^−/−^ melanoma tumor implanted in *Carmil2*^QE^*Cd28*^−/−^ mice.

The T_reg_ cells present in *Carmil2*^QE^*Cd28*^−/−^ mice had a twofold decreased suppressive activity as compared to their WT counterpart (Fig. 5 C), and it might have also contributed to the capacity of *Carmil2*^QE^*Cd28*^−/−^ mice to eradicate BRAF^V600E^*Ptgs*^−/−^ tumors. Therefore, to evaluate whether the expression of the *Carmil2*^QE^ mutation in and only in CD8^+^ T cells sufficed to mount protective anticancer responses, we analyzed the growth of the syngeneic MC38-OVA colon adenocarcinoma tumor transplanted subcutaneously into WT mice with or without adoptive transfer of OT-I or OT-I *Carmil2*^QE^ naive T cells. Considering that MC38-OVA is sensitive to the PD-1 immune checkpoint inhibitor, cohorts of tumor-bearing mice were also injected with anti-PD-1 or with isotype control antibodies to serve as positive and negative controls, respectively (Fig. 8, C and D). In contrast to OT-I naive T cells, OT-I *Carmil2*^QE^ naive T cells rejected MC38-OVA tumors and the levels of rejection achieved were similar to those observed in tumor-bearing WT mice treated with anti-PD-1. Comparison of the immune cells infiltrating MC38-OVA tumors 11 days after inoculation showed that the MC38-OVA tumors from mice that have received OT-I *Carmil2*^QE^ T cells contained increased numbers of OT-I *Carmil2*^QE^ T cells and reduced numbers of host T_reg_ cells and NK cells as compared to MC38-OVA tumors isolated from mice that have received WT OT-I T cells (Fig. 8 E). Taken together, these results demonstrate that the expression of the *Carmil2*^QE^ mutation in and only in cancer-specific CD8^+^ T cells sufficed to trigger their intratumoral expansion, resulting in protective T cell–mediated anticancer immunity without the need for anti-PD-1 therapy.

### Composition and dynamics of the CARMIL2 and CARMIL2^QE^ interactomes

**Figure S4.**
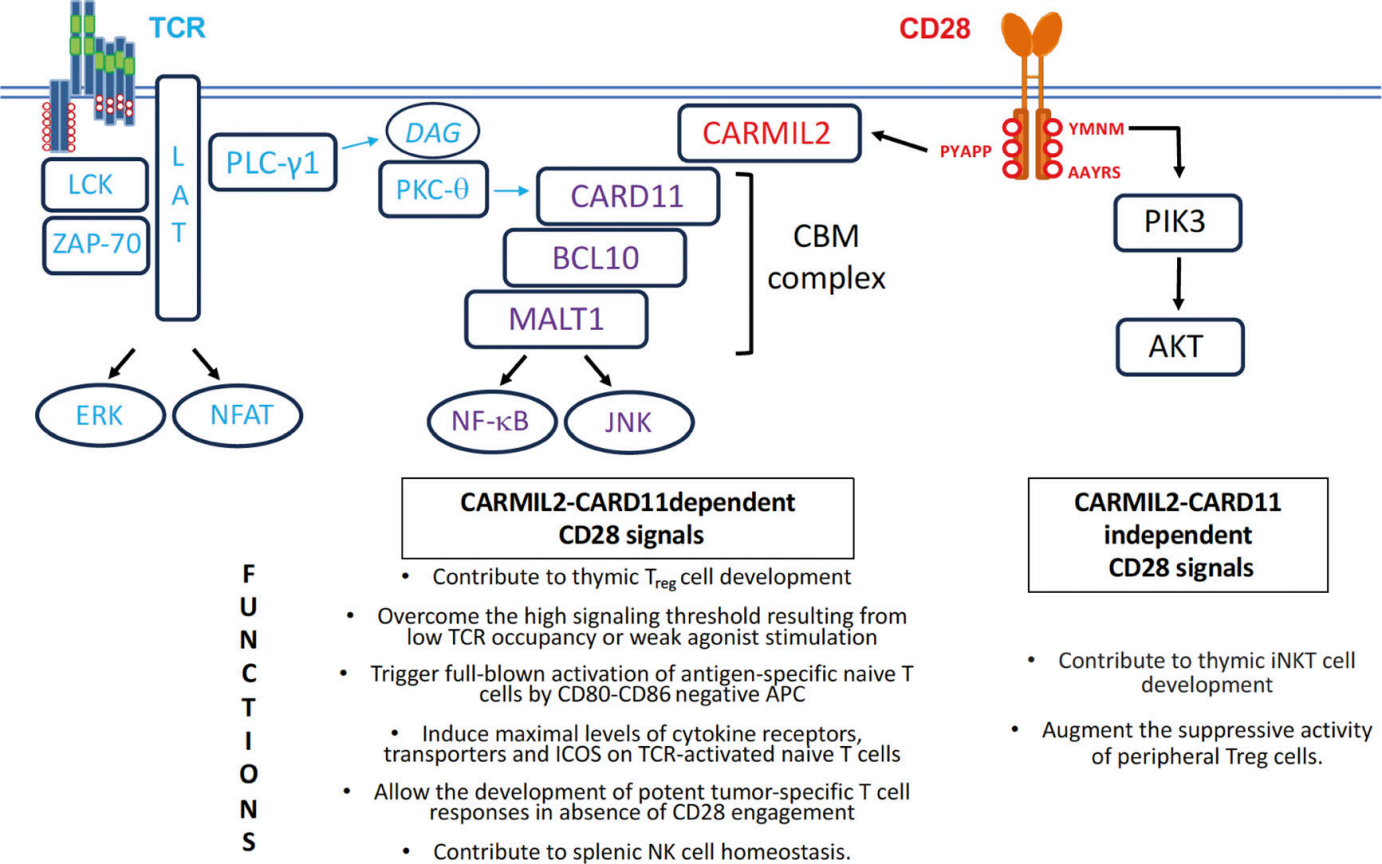
**Model summarizing the CD28-dependent traits induced by CARMIL2-CARD11-dependent or CARMIL2-CARD11-independent CD28 signals.** In naive T cells, CD28 engagement by its CD80-CD86 ligands expressed on immunogenic APC triggers both CARMIL2-CARD11-dependent and CARMIL2-CARD11-independent signaling branches. The developmental and functional consequences of CD28 engagement for which the CARMIL2-CARD11-dependent or CARMIL2-CARD11-independent CD28 signaling branches are necessary and sufficient are shown below each signaling branch. Engagement of the TCR results in the activation of the LCK and ZAP-70 protein tyrosine kinases. It leads to the formation of the LAT signalosome, which controls phospholipase PLC-γ1 activity and triggers the production of inositol 1,4,5-trisphosphate and diacylglycerol (DAG), ultimately resulting in the activation of the NFAT and AP1 transcription factors (Shapiro et al., 1998). DAG also promotes the recruitment of the protein serine/threonine kinase PKC-θ at the plasma membrane, enabling its incorporation into the CD28 microclusters that form at the immune synapse and contain CARMIL2-CARD11 complexes (Liang et al., 2013). Following phosphorylation by PKC-θ and additional TCR-operated protein serine/threonine kinases, the CARD11 molecules bound to CARMIL2 associate with BCL10 and MALT1 to give rise to CBM complexes capable of activating the NF-κB transcription factor, the JNK, and the MALT1 protease (Ruland and Hartjes, 2019). PI3K constitutes one of the effectors of the CARMIL2-CARD11-independent CD28 signaling branch. It accounts for CD28-mediated production of phosphatidylinositol (3,4,5)-triphosphate (PIP3), leading to the recruitment and activation of the PH domain–containing protein kinase AKT and the occurrence of CD28-CD80 cis interactions at the immune synapse (Zhao et al., 2023). CD28-generated PIP3 also enhances the recruitment and activity of ITK and PLC-γ1, two pleckstrin homology domain–containing proteins that are part of the TCR-operated LAT signalosome, thereby reinforcing the production of DAG (Michel et al., 2001). CD28 costimulation also promotes the ubiquitylation and proteasomal degradation of the E3 ubiquitin ligase CBL-B, and regulates mRNA processing and T cell metabolism via signals that remain to be characterized (Lotze et al., 2024). The CD28 cytoplasmic tail contains three distinct protein–protein interaction motifs denoted as YMNM, PYAPP, and AAYRS (red circles), and the PYAPP motif is the sole necessary to trigger the CARMIL2-CARD11-mediated signals; JNK, c-Jun N-terminal kinase.

Upon TCR-CD28 stimulation, several proteins interact with CARMIL2 in addition to CARD11 to form a multiprotein complex denoted as the CARMIL2 interactome or signalosome (Roncagalli et al., 2016). To help rationalizing the unexpectedly broad in vivo functional consequences of the *Carmil2*^QE^ mutation (summarized in Fig. S4), we analyzed whether the *Carmil2*^QE^ mutation affected other CARMIL2-interacting partners beyond CARD11. Using affinity purification coupled with mass spectrometry (AP-MS; Voisinne et al., 2019), we compared the composition of the CARMIL2 and CARMIL2^QE^ interactomes of mouse CD4^+^ T cells isolated from *Carmil2*^OST^ and *Carmil2*^QE-OST^ mice prior to and following T cell activation (Table S2 and Data S1). Both interactomes contained CD28, CARD11, and PKC-θ (coded by the *Prkcq* gene), demonstrating that CARMIL2^QE^ molecules remained capable of complexing CD28 to both CARD11 and PKC-θ (Liang et al., 2013; Roncagalli et al., 2016). CAPZA2 and CAPZB, which correspond to the α- and β-subunits of actin capping protein and constitutively bind to CARMIL2 (Stark et al., 2017), were also found in both interactomes. Likewise, both interactomes contained the isoform alpha of casein kinase 1 (CK1-α coded by the *Csnk1a1* gene), the serine/threonine protein kinase MAP4K1 (also known as HPK1), the calcium–calmodulin-dependent protein kinase II delta (CAMK2D), and the deubiquitinase ubiquitin carboxyl-terminal hydrolase 9X (USP9X), which, together with PKC-θ, collectively regulate the assembly and signaling output of CBM complexes via phosphorylation events (Bidère et al., 2009; Gehring et al., 2019; Kutzner et al., 2022; Park et al., 2013). Several additional interacting proteins, the role of which remains to be elucidated, were also present in both the CARMIL2 and CARMIL2^QE^ interactomes (Table S2 and Data S1). Therefore, the *Carmil2*^QE^ mutation does not modify the global composition of the WT CARMIL2 interactome.

**Figure S5.**
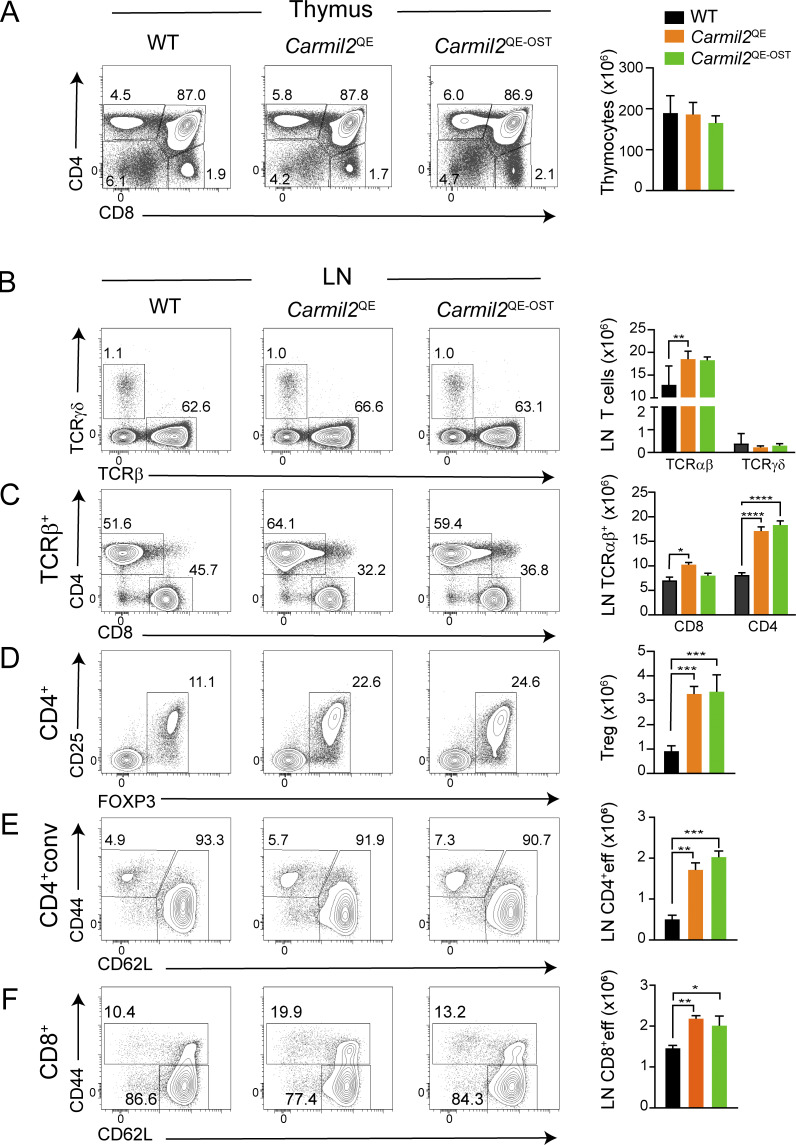
**Thymus and LN of WT, *Carmil2*** ^**QE**^ **, and *Carmil2*** ^**QE-OST**^ **mice have similar phenotypes. (A)** WT, *Carmil2*^QE^, and *Carmil2*^QE-OST^ thymi were analyzed by flow cytometry for the expression of CD4 and CD8. Numbers indicate the percentage of CD4^−^CD8^−^ double-negative, CD4^+^CD8^+^ double-positive, and CD4^+^ and CD8^+^ single-positive cells. Also shown on the right is the cellularity of WT, *Carmil2*^QE^, and *Carmil2*^QE-OST^ thymi. **(B)** Total cells from WT, *Carmil2*^QE^, and *Carmil2*^QE-OST^ LN were analyzed by flow cytometry for the expression of TCRβ and TCRγδ, and the corresponding percentages of TCRβ^+^ and TCRγδ^+^ cells were indicated by the number adjacent to outlined areas. Also shown on the right is the number of TCRβ^+^ and TCRγδ^+^ cells in WT, *Carmil2*^QE^, and *Carmil2*^QE-OST^ LN (see key in A). **(C)** TCRβ^+^ cells from WT, *Carmil2*^QE^, and *Carmil2*^QE-OST^ LN (see gating strategy in B) were analyzed for CD4 and CD8 expression and the percentages of CD4^+^ and CD8^+^ cells indicated by the number adjacent to outlined areas. Also shown on the right is the number of TCRαβ^+^ CD4^+^ and CD8^+^ Τ cells in WT, *Carmil2*^QE^, and *Carmil2*^QE-OST^ LN (see key in A). **(D)** FOXP3^+^ T_reg_ cells present among total CD4^+^ T cells isolated from WT, *Carmil2*^QE^, and *Carmil2*^QE-OST^ LN were analyzed by flow cytometry using FOXP3 and CD25 expression, and their percentages were indicated by the number adjacent to outlined areas. Also shown on the right is the number of FOXP3^+^ T_reg_ cells in WT, *Carmil2*^QE^, and *Carmil2*^QE-OST^ LN (see key in A). **(E)** CD4^+^ T_conv_ cells found in the LN of WT, *Carmil2*^QE^, and *Carmil2*^QE-OST^ mice (gated as in D) were analyzed by flow cytometry using CD44 and CD62L expression, and the percentages of naive (CD44^lo^CD62L^hi^) and effector-memory (CD44^hi^CD62L^lo^) cells were indicated by the number adjacent to outlined areas. Also shown on the right is the number of effector-memory CD4^+^ T cells in WT, *Carmil2*^QE^, and *Carmil2*^QE-OST^ LN (see key in A). **(F)** CD8^+^ T cells found in the LN of WT, *Carmil2*^QE^, and *Carmil2*^QE-OST^ mice (gated as in C) were analyzed by flow cytometry using CD44 and CD62L expression, and the percentages of naive (CD44^lo^CD62L^hi^) and central plus effector-memory (CD44^hi^CD62L^low to high^) cells were indicated by the number adjacent to outlined areas. Also shown on the right is the number of central plus effector-memory CD8^+^ T cells in WT, *Carmil2*^QE^, and *Carmil2*^QE-OST^ LN (see key in A). Data in A–D are representative of two independent experiments, with two mice per genotype.

**Figure 9.**
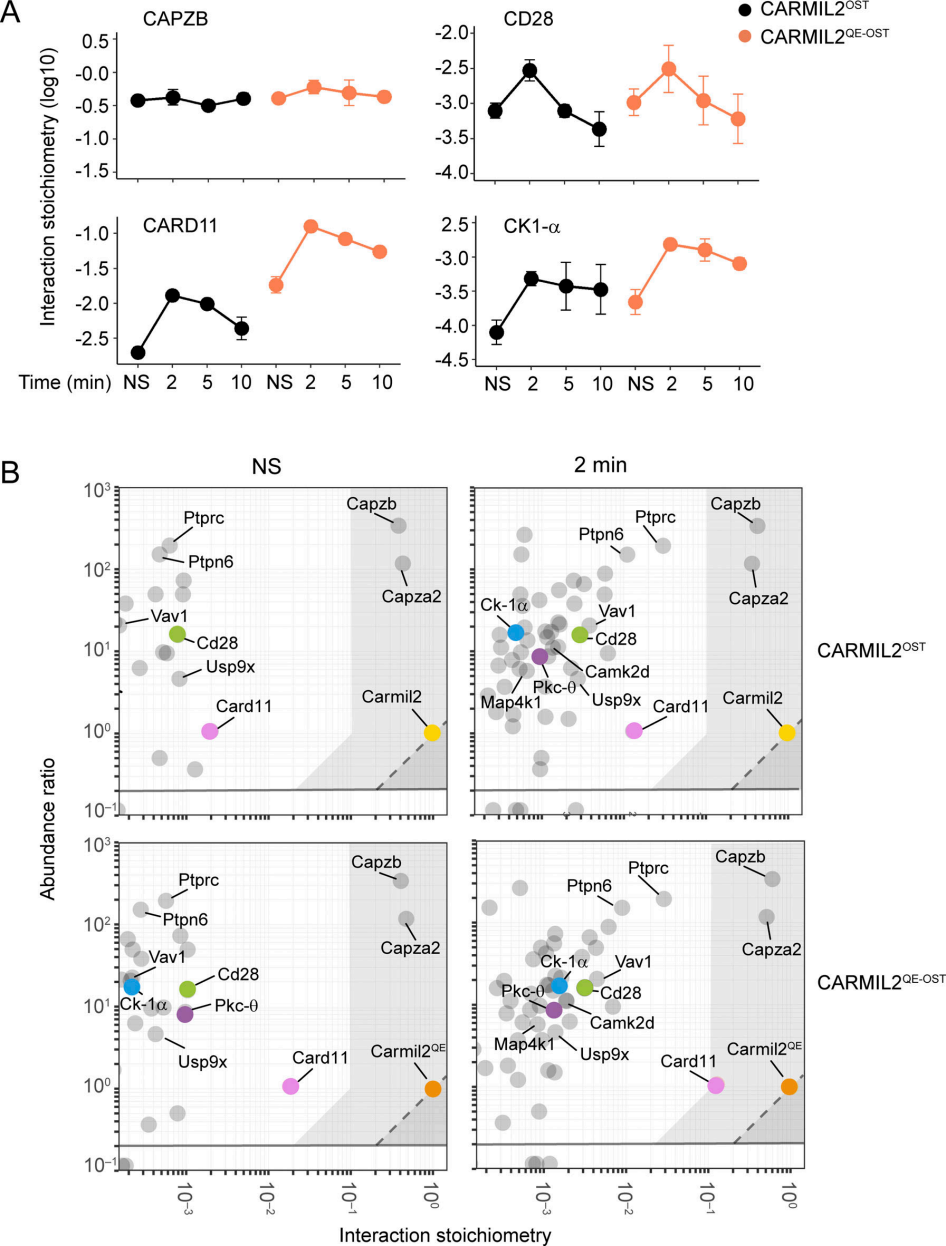
**Composition, dynamics, and stoichiometry of the CARMIL2 and CARMIL2** ^**QE**^ **interactomes of primary mouse CD4** ^**+**^ **T cells. (A)** Plots showing the interaction stoichiometry (in log_10_ scale) of CARMIL2^OST^ and CARMIL2^QE-OST^ molecules with the CAPZB-, CD28-, CARD11-, and CK1-α–interacting proteins in CD4^+^ T cells before (NS = not stimulated) and after 2, 5, and 10 min of activation via pervanadate treatment. The CARMIL2^QE-OST^-CK1-α bait–prey interaction was the sole to show a temporal pattern of interaction stoichiometry similar to that of CARMIL2 ^QE-OST^-CARD11. Data are representative of three independent experiments each involving three replicates (mean ± SEM; *n* = 9 for each time point). **(B)** Stoichiometry plots of the CARMIL2^OST^ and CARMIL2 ^QE-OST^ interactome in CD4^+^ T cells prior to activation (NS) and after 2 min of activation (see Data S1). The CARMIL2^OST^ (yellow dots) and CARMIL2^QE-OST^ (orange dots) proteins (corresponding to the two “baits”), and the CD28 (green dots)-, CARD11 (pink dots)-, PKC-θ (purple dots)–, and CK1-α (blue dots)–interacting proteins (corresponding to select high-confidence “preys”) are highlighted, whereas the remaining high-confidence preys are shown as gray dots. For each of the bait–prey interactions, the ratio of prey to bait cellular abundance (abundance ratio in the log_10_ scale) was plotted as a function of the interaction stoichiometry of the considered bait–prey interaction (interaction stoichiometry in the log_10_ scale). As already noted in the case of the TCR signaling network (Voisinne et al., 2019), substoichiometric bait–prey interactions play a central role in the organization of the CARMIL2^OST^ and CARMIL2^QE-OST^ interactome. The two exceptions corresponded to the almost stoichiometric bait–prey interactions involving CARMIL2^OST^ and CARMIL2^QE-OST^ with CAPZB and CAPZA2 and to the maximal interaction stoichiometry reached after 2 min of activation by the CARMIL2^QE-OST^-CARD11 bait–prey interaction, a condition in which 12% of the available CARMIL^QE-OST^ molecules are complexed to CARD11. The area corresponding to bait–prey interaction involving >10% of the available prey molecules is indicated in light gray and includes CAPZA2 and CAPZB in all the analyzed conditions, and CARD11 in the case of the CARMIL2^QE-OST^ interactome after 2 min of stimulation.

Considering that immunoblots provide limited quantitative insights, we used quantitative AP-MS to determine the interaction stoichiometries (Schwanhäusser et al., 2011; Voisinne et al., 2019) of CARMIL2 and CARMIL2^QE^ with each of their high-confidence interacting partners over 10 min of T cell activation. These interaction stoichiometries showed three distinct temporal profiles. First, both CARMIL2 and CARMIL2^QE^ constitutively associated with CAPZA2 and CAPZB with similar high interaction stoichiometry (Fig. 9 A and Data S1). Second, most of the identified interacting partners required prior T cell activation to associate with both CARMIL2 and CARMIL2^QE^ molecules and showed similar low interaction stoichiometries (illustrated using CD28 in Fig. 9 A). A third temporal profile of interaction stoichiometries involved CARD11 and differed between the CARMIL2 and CARMIL2^QE^ interactomes. Consistent with our biochemical analysis (Fig. 2), the interaction between CARMIL2 and CARD11 molecules required prior activation and its stoichiometry peaked 2 min after activation (Fig. 9 A). In contrast, a fraction of CARMIL2^QE^ proteins were already associated with CARD11 proteins in resting T cells and showed an interaction stoichiometry comparable to the maximal CARMIL2-CARD11 interaction stoichiometry observed in WT T cells 2 min after T cell activation (Fig. 9 A). Moreover, after T cell activation, the CARMIL2^QE^-CARD11 interaction stoichiometry increased sevenfold above that observed in resting CARMIL2^QE^ CD4^+^ T cells. Analysis of the correlations between all the temporal profiles of interaction stoichiometries further revealed that CK1-α was the only interacting partner whose recruitment to CARMIL2 and CARMIL2^QE^ correlated with that of CARD11 (Fig. 9 A and Data S2). However, the maximal interaction stoichiometries reached by CARMIL2-CK1-α and CARMIL2^QE^-CK1-α remained 24- and 80-fold lower than those of CARMIL2-CARD11 and CARMIL2^QE^-CARD11, respectively (Data S1). The absence of interactor displaying both a temporal profile of interaction stoichiometry and an interaction stoichiometry similar to those of the CARMIL2-CARD11 and CARMIL2^QE^-CARD11 interactions strongly suggests that CARMIL2 and CARMIL2^QE^ molecules directly interact with CARD11 molecules without the need for a bridging protein. Therefore, once the CARD11- and CK1-α–interacting partners are set aside, the *Carmil2*^QE^ mutation does not change the temporal profile of interaction stoichiometries of the remaining interacting proteins found in the WT CARMIL2 interactome.

### Systems-level view of the molecular consequences of the *Carmil2*^QE^ mutation

**Figure 10.**
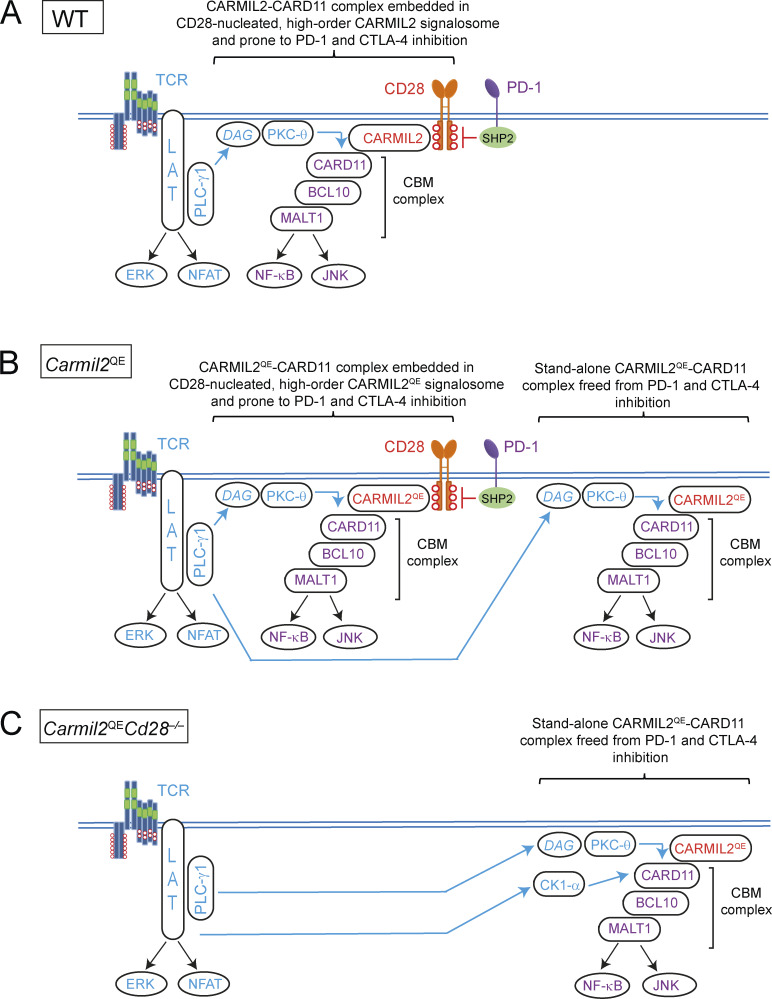
**Model summarizing the mode of action of CARMIL2 and CARMIL2** ^**QE**^ **molecules in T cells expressing or lacking CD28. (A)** CARMIL2 molecules function as CD28-inducible scaffolds that recruit the CARD11 adaptor. All the CD28-inducible CARMIL2-CARD11 complexes that form in WT T cells are embedded within CD28-nucleated, high-order CARMIL2 signalosome. Phosphorylation of the CARMIL2-associated CARD11 molecules by TCR-activated protein serine/threonine kinases that include PKC-θ induces the formation of active CBM complexes that trigger downstream signaling events including the activation of the NF-κB transcription factor (see Fig. S4 and Bidère et al., 2009; Gehring et al., 2019; Kutzner et al., 2022; Liang et al., 2013; Park et al., 2013; Roncagalli et al., 2016; Schober et al., 2017). The tyrosine-based protein–protein interaction motifs (red circles) present in the CD28 intracytoplasmic tail are subjected to dephosphorylation by the SHP2 protein tyrosine phosphatase associated with the PD-1 coinhibitor (Celis-Gutierrez et al., 2019). The CD80 and CD86 ligands expressed at the surface of APC are also subjected to CTLA-4–mediated T_reg_ cell transendocytosis (not shown). **(B)** Majority of the CARMIL2^QE^-CARD11 complexes found in activated *Carmil2*^QE^ T cells lie outside of CD28-nucleated, high-order CARMIL2 signalosomes and are denoted as stand-alone CARMIL2^QE^-CARD11 complexes. The activity of these last complexes remains dependent on TCR inputs (blue arrow) and freed from PD-1 and CTLA-4 inhibition. **(C)** Stand-alone CARMIL2^QE^-CARD11 complexes are the sole to form in *Carmil2*^QE^*Cd28*^−/−^ T cells. Following phosphorylation by TCR-operated serine/threonine kinases, they likely constitute the seed of the functional CBM complexes that mediate most of the functions attributed to CD28 in *Carmil2*^QE^*Cd28*^−/−^ T cells. The activity of these stand-alone CARMIL2^QE^-CARD11 complexes remains dependent on TCR inputs (blue arrow) and freed from PD-1 and CTLA-4 inhibition. The interaction between CARMIL2^QE^ and isoform CK1α shows a temporal profile of interaction stoichiometry similar to that of the CARMIL2^QE^-CARD11 interaction. Considering that CK1α is essential for CBM assembly and MALT1 phosphorylation (Bidère et al., 2009; Gehring et al., 2019), it suggests that the TCR-triggered activation signals received by the stand-alone CARMIL2^QE^-CARD11 complexes are mediated at least by CK1α.

To quantitatively compare at a glance the CARMIL2^OST^ and CARMIL2^QE^ interactomes that form in CD4^+^ T cells after 2 min of activation, we combined the interaction stoichiometries of CARMIL2 and CARMIL2^QE^ molecules with each of their interacting partners together with their respective cellular abundance (numbers of copies per CD4^+^ T cells; Data S1) (Voisinne et al., 2019). The resulting stoichiometry plots showed that aside of the interactions involving CARD11 and CK1-α, all the other high-confidence protein–protein interactions occupied a similar position on the CARMIL2 and CARMIL2^QE^ stoichiometry plots at 2 min of activation, confirming the globally conserved composition and dynamics of the CARMIL2 and CARMIL2^QE^ interactomes (Fig. 9 B). Therefore, both WT and *Carmil2*^QE^-activated T cells contained identical CD28-nucleated, high-order CARMIL2 and CARMIL2^QE^ signalosomes that reached comparable numbers of copies per T cell after 2 min of activation (Fig. 9 B). However, additional CARMIL2^QE^-CARD11 complexes specifically formed in *Carmil2*^QE^-activated T cells independently of the CD28-nucleated, high-order CARMIL2^QE^ signalosomes. Such “stand-alone” CARMIL2^QE^-CARD11 complexes were 10-fold more numerous than those embedded in the CD28-nucleated, high-order CARMIL2^QE^ signalosomes. They readily formed in the absence of CD28 as documented in *Carmil2*^QE^*Cd28*^−/−^ T cells (Fig. 2), and following phosphorylation by TCR-operated serine/threonine kinases likely constitute the seed of the functional CBM complexes that are capable of replacing the need for CD28 in *Carmil2*^QE^*Cd28*^−/−^ T cells (Fig. 10).

## Discussion

The CD28 cytoplasmic tail contains tyrosine- and proline-based protein–protein interaction motifs that recruit in a direct or indirect manner a series of proteins that includes, among others, phosphatidylinositol 3-kinase (PI3K), the GRB2 adaptor, the LCK and PKC-θ protein kinases (Chen et al., 2022; Skånland and Taskén, 2019; Tian et al., 2015), and CARMIL2 (Liang et al., 2013). Each of these proteins is thought to add by itself or in combination a specific functionality to CD28. Using CD28-deficient mice expressing a *Carmil2*^QE^ mutation, we demonstrated here the unanticipated ability of CARMIL2-CARD11-mediated CD28 signals to trigger most known CD28 costimulatory functions in vivo independently of other CD28 signals. Considering the important role attributed to PI3K during CD28 costimulation (Michel et al., 2001), it appears paradoxical that *Carmil2*^Q578E^*Cd28*^−/−^ mice developed robust naive T cell activation and antitumor responses in the absence of CD28-mediated PI3K signals. This might have resulted from the expression on naive *Carmil2*^QE^*Cd28*^−/−^ T cells of receptors capable of replacing CD28-triggered PI3K signals. CD28 and ICOS are evolutionary-related costimulatory receptors that both induce PI3K activation (Fos et al., 2008). In mice carrying a homozygous mutation in the gene coding for ROQUIN-1, ICOS is abnormally expressed on naive CD4^+^ and CD8^+^ T cells and can substitute in part for the missing CD28-mediated PI3K signals (Linterman et al., 2009). In contrast, ICOS was absent on *Carmil2*^QE^*Cd28*^−/−^ naive T cells and only induced after activation, demonstrating that it cannot provide redundant PI3K signals to *Carmil2*^QE^*Cd28*^−/−^ naive T cells at the onset of their activation.

Interestingly, the capacity to deliver CARMIL2-CARD11-dependent or CARMIL2-CARD11-independent CD28 signals correlated with the use of distinct protein–protein interaction motifs in the CD28 cytoplasmic tail. For instance, the PYAP motif is mandatory for the generation of normal numbers of thymic T_reg_ cells, optimal activation of naive T_conv_ cells, and IL-2 production (Dodson et al., 2009; Friend et al., 2006; Tai et al., 2005), three outcomes for which we showed that CARMIL2-CARD11-mediated CD28 signals were necessary and sufficient to replace CD28. Conversely, iNKT cell generation correlated with the use of CD28 cytoplasmic motifs distinct from the PYAP motif and required CARMIL2-CARD11-independent CD28 signals (Watanabe et al., 2022). These correlations are congruent with the view that CARMIL2 (this study) and the CD28 PYAP motif (Boomer et al., 2014; Chan et al., 2023; Dobbins et al., 2016; Friend et al., 2006; Garçon et al., 2008; Lotze et al., 2024; Pagán et al., 2012; Tai et al., 2007; Watanabe et al., 2020) are essential to induce most of the CD28-dependent developmental and functional outcomes.

CARMIL2 molecules function as scaffolds permitting the CD28-inducible assembly of CARMIL2-CARD11 complexes. Following phosphorylation by TCR-operated serine/threonine kinases, those complexes constitute the seed permitting the formation of active CBM complexes. After activation of WT T cells, all the CD28-nucleated CARMIL2-CARD11 complexes that assemble are embedded within high-order CARMIL2 interactomes (Fig. 10). In contrast, in activated *Carmil2*^QE^ T cells the majority of CARMIL2^QE^-CARD11 complexes assemble independently of CD28-nucleated, high-order CARMIL2^QE^ signalosomes. Moreover, such stand-alone CARMIL2^QE^-CARD11 complexes are the sole to form in *Carmil2*^QE^*Cd28*^−/−^ T cells. Following phosphorylation by TCR-operated serine/threonine kinases, these stand-alone CARMIL2^QE^-CARD11 complexes likely constitute the seed of the active CBM complexes that mediate in *Carmil2*^QE^*Cd28*^−/−^ mice most of the functions attributed to CD28. Therefore, our quantitative interactomics and functional analyses permit to qualify the *Carmil2*^QE^ mutation as a genuine gain-of-function mutation due to its ability to induce the assembly of CARMIL2^QE^-CARD11 complexes in the absence of CD28 input. Mechanistically, the *Carmil2*^QE^ mutation might cause a conformational change in CARMIL2 proteins similar to the one that likely happens during their physiological interaction with CD28 molecules bound to CD80-CD86 ligands, and which increases their affinity for CARD11. It also explained that the effects of the *Carmil2*^QE^ mutation remained dependent on TCR-triggered posttranslational modifications of CARMIL2^QE^-bound CARD11 molecules (Kutzner et al., 2022). The functional consequence of this selective gain-of-function in CARD11 recruitment in mice is to correct the majority of activation and differentiation defects caused by CD28 deficiency or by the absence of the CD80 and CD86 ligands including T_reg_ cell development and tumor eradication. However, a subset of CD28-dependent events, notably invariant NKT cell development in the thymus, are not CARMIL2-dependent and in turn not corrected by the *Carmil2*^QE^ mutation.

In the two-signal model of naive T cell activation, signal 2 is delivered by a triggering module made of CD28 and of its CD80-CD86 ligands and is conveyed via protein–protein interaction motifs that activate either CARMIL2-CARD11-dependent or -independent signaling branches. The CD28 triggering module of naive T cells is negatively controlled by mechanisms, which are T cell–intrinsic and involve PD-1–mediated dephosphorylation of the tyrosine-based CD28 protein–protein interaction motifs (Celis-Gutierrez et al., 2019; Chan et al., 2023; Hui et al., 2017), as well as T cell–extrinsic and rely on CTLA-4–mediated T_reg_ cell transendocytosis of CD80 and CD86 at the surface of APC (Ovcinnikovs et al., 2019). As a result, T cell reactivity against self- and foreign peptides is tuned down by both the action PD-1 and CTLA-4 and the dynamic changes in CD80, CD86, and PD-L1 expression occurring on APC during their tolerogenic or immunogenic maturation (Ardouin et al., 2016; Brown and Rudensky, 2023). Under physiological conditions, these negative regulatory mechanisms permit to eliminate disease-causing pathogens without damaging body tissues and tumors have exploited them for immune escape (Agarwal et al., 2023; Duraiswamy et al., 2021; Magen et al., 2023). We showed here that the ready-made, stand-alone CARMIL2^QE^-CARD11 complexes found in naive *Carmil2*^QE^*Cd28*^−/−^ T cells replaced the need for a CD28 triggering module. It coincidentally allowed *Carmil2*^QE^*Cd28*^−/−^ T cells to escape the inhibitory effects resulting from both PD-1 engagement and T_reg_ cell–mediated CD80-CD86 transendocytosis, and likely contributed to the unique ability of *Carmil2*^QE^ OT-I T cells to achieve complete rejection of MC38-OVA tumors in the absence of anti-PD-1 treatment.

The separate positive signaling inputs provided by the TCR and CD28 converge on the CARD11 molecule (Fig. 10). Therefore, CARD11 functions as a coincidence detector that informs T cells that their TCR interacted with antigen-laden APC that have been subjected to inflammatory cues and express high levels of CD80-CD86, leading to their differentiation into effector T cells. Considering that self-reactive T cells can emerge from the thymus (Yu et al., 2015), the *Carmil2*^QE^ mutation should have freed them from both PD-1– and T_reg_ cell–mediated inhibition and lead them to escape peripheral tolerance mechanisms and in turn differentiate into effector T cells capable of triggering autoimmunity. Although up to fourfold increased numbers of central and effector-memory CD4^+^ T_conv_ and CD8^+^ T cells were found in LN of 10-wk-old *Carmil2*^QE^*Cd28*^−/−^ mice as compared to age-matched WT mice, cohorts of *Carmil2*^QE^*Cd28*^−/−^ mice maintained over a period of 50 wk under specific pathogen-free conditions showed no obvious sign of autoimmune pathologies and had weight gain and survival rate comparable to age-matched WT mice. The absence of blatant autoimmunity signs in *Carmil2*^QE^*Cd28*^−/−^ mice kept under specific pathogen-free conditions suggests that their naive T cells expressing high-affinity self-reactive TCR remain subjected to additional peripheral tolerance mechanisms not relying on PD-1– and transendocytosis-based T_reg_ cell–mediated suppressive mechanisms (Brown and Rudensky, 2023; Gronski et al., 2004; Policheni et al., 2022; Wong et al., 2021). Accordingly, *Carmil2*^QE^*Cd28*^−/−^ self-reactive T cells might further require CARMIL2-CARD11-independent CD28 signals and exposure to APC-derived pro-inflammatory cytokines to trigger autoimmune manifestations. Finally, *Carmil2*^QE^*Cd28*^−/−^ mice maintained over a period of 50 wk developed no malignancy or lymphoproliferative disorder, indicating that the *Carmil2*^QE^ mutation does not constitute by itself a cancer driver mutation and likely manifests its gain-of-function effects only in the context of the additional oncogenic mutations found in T cell lymphoma and leukemia (Park et al., 2017; Uchida et al., 2021).

In conclusion, our study demonstrates that most of the developmental and functional consequences resulting from CD28 costimulation are induced by CARMIL2-CARD11-mediated signals and emphasizes the overarching role played by those signals among those triggered by CD28. It also illustrates the power of quantitative interactomics to disentangle the mechanism of action a given mutation has on a multiprotein signalosome, a possibility used here to demonstrate that the change induced in the sole CARMIL2-CARD11 interaction accounted for the unexpectedly broad in vivo functional effects of the *Carmil2*^QE^ mutation. Finally, our results concur with a recent study (Garcia et al., 2024) to demonstrate that among the mutations occurring in malignant T cells, those, the effects of which remain dependent on antigen-dependent TCR signals, can be harnessed to enhance the efficacy of therapeutic T cells. Along that line, we showed that the unique properties of CARMIL2^QE^ molecules could be exploited to enhance the efficacy of therapeutic T cells in environment deprived of CD28 ligands and to concomitantly free them from PD-1 and CTLA-4 inhibition.

## Materials and methods

### Mice

Mice were on a C57BL/6 (B6) background and 8–12 wk old unless specified. They were maintained under specific pathogen-free conditions at Centre d’Immunophénomique (accreditation B1301407) and Centre d’Immunologie de Marseille-Luminy (accreditation F13005). OT-I (Hogquist et al., 1994), *Cd28*^−/−^ (Shahinian et al., 1993), *Cd80*^−/−^*Cd86*^−/−^ (Borriello et al., 1997), *Cd3ε*^Δ5/Δ5^ and Cd3ε^Δ5/Δ5^*Cd80*^−/−^*Cd86*^−/−^ (Malissen et al., 1995) mice have been described. Control mice correspond to mice of the same genetic background raised in the same animal facility.

### Animal experimental guidelines

Mice were handled in accordance with national and European laws for laboratory animal welfare and experimentation (European Economic Community Council Directive 2010/63/EU, September 2010) and protocols approved by the Marseille Ethical Committee for Animal Experimentation. The generation of knock-in mouse expressing *Carmil2*^Q538E^ and *Carmil2*^Q538E-OST^ alleles was performed in accordance with Xinxiang Medical University (Xinxiang, China) guidelines for animal care.

### Generation of knock-in mouse expressing a *Carmil2*^OST^ allele

A targeting vector was designed to introduce a nucleotide sequence coding for a Twin-Strep-tag (5′-ASWSHPQFEKGGGSGGGSGGGSWSHPQFEK-3′) and a Gly-Gly-Ala amino acid spacer between the first (ATG) and the second (GCA) codon of the mouse *Carmil2* gene (ENSMUST00000213019.2 Carmil2-203). A self-excising ACN cassette was introduced in the intron located between *Carmil2* exons 1 and 2, and a cassette permitting the expression of a diphtheria toxin fragment was abutted to the targeting construct. JM8.F6 B6N ES cells were electroporated with the targeting vector. After selection in G418, ES cell clones were screened for proper homologous recombination by Southern blot and PCR analysis. A probe specific for the neo^r^ cassette was further used to ensure that adventitious nonhomologous recombination events had not occurred in the selected clones. Mutant ES cells were injected into FVB blastocysts. Screening for proper autodeletion of the ACN cassette and for the presence of the sequence coding for the Twin-Strep-tag (abbreviated as OST) was performed by PCR and sequencing. The resulting mutant mice are denoted as *Carmil2*^OST^ mice and also known as B6-*Rltpr*^tm3Mal^. Genotyping of the *Carmil2*^OST^ allele was performed by PCR using two pairs of primers. The first pair (sense 5′-CTG​GCT​TCC​TGT​GTA​CGC​TC-3′ and antisense 5′-ACC​TGG​TGA​TCT​CGC​CTG​TG-3′) amplified a 369-bp band in the case of the WT allele, whereas the second pair (sense 5′-AGA​TCT​CGA​GCT​CGC​GAA​AG-3′ and antisense 5′-ACC​TGG​TGA​TCT​CGC​CTG​TG-3′) amplified a 233-bp band in the case of the *Carmil2*^OST^ allele.

### Generation of knock-in mouse expressing a *Carmil2*^Q538E^ allele

A homology-directed repair (HDR) template consisting of a single-stranded oligonucleotide (BiOligo Biotechnology Co., Ltd; Table S3) was designed to convert the CAG codon found in exon 20 of the mouse *Carmil2* gene and coding for the glutamine residue present at position 538 of the CARMIL2 protein into a GAG codon coding for a glutamic acid. It was used together with a single guide RNA (sgRNA; Table S4) targeting exon 20 of the *Carmil2* gene. Fertilized eggs from B6 female were microinjected with Cas9 mRNA and the designed sgRNA and HDR template as described previously (Zhang et al., 2022). Tail genomic DNA was isolated from the resulting F0 mice and the region encompassing exon 20 amplified and sequenced. An F0 mouse expressing the intended Q538E mutation was used to establish mice heterozygous and homozygous for the Q538E mutation. Those mice denoted as *Carmil*^Q538E^ or *Carmil*^QE^ in short are also known as B6-*Rltpr*^tm4Mal^. *Carmil2*^Q538E^ mice were genotyped by sequencing a 509-bp DNA fragment that was amplified using the following pair of PCR primers: sense 5′-GAC​ATG​GTG​ACA​CTG​GTG​CT-3′ and antisense 5′-GAG​CCT​TGG​CTA​GCA​TCT​TG-3′.

### Generation of knock-in mouse expressing a *Carmil2*^Q538E-OST^ allele

An HDR template consisting of a single-stranded DNA (BiOligo Biotechnology Co., Ltd.; Table S3) was designed to insert a sequence coding for an improved Twin-Strep-tag (5′-SAWSHPQFEKGGGSGGGSGGSAWSHPQFEK-3′) and a GSG spacer between the first (ATG) and the second (GCA) codon of the first exon of the *Carmil2*^Q538E^ allele. It was used together with two sgRNAs targeting the ATG start codon (Table S4). Fertilized eggs from B6 female mice homozygous for the *Carmil2*^Q538E^ allele were microinjected with Cas9 mRNA and the designed sgRNA and HDR template as described above. Tail genomic DNA was isolated from the resulting F0 mice and the region encompassing exon 1 amplified and then sequenced. An F0 mouse expressing the intended Twin-Strep-Tag insertion was used to establish homozygous mutant mice. Those mice denoted as *Carmil2*^Q538E-OST^ or *Carmil2*^QE-OST^ in short are also known as B6-Carmil2^tm5Mal^. *Carmil2*^Q538E-OST^ mice were genotyped by using the following pair of PCR primers: sense 5′-ATT​CGA​CCA​TCC​TCC​CAC​AAC-3′ and antisense 5′-GAG​AGG​TCT​GGT​TTG​GAG​TCA​G-3′. They amplified a 413 and 314-bp band in the case of the *Carmil2*^Q538E-OST^ and WT *Carmil2* allele, respectively.

### Thymus, LN, and spleen cell preparations

Cells from thymus, spleen, and LN were prepared by mechanical disruption in RPMI medium containing 2% FCS. Red blood cells were lysed using RBC lysis buffer (eBioscience). Single-cell suspensions were filtered through a 100-μm membrane prior to counting.

### Flow cytometry

The following antibodies were used: anti-CD3 (145-2C11), anti-CD4 (RMA-5), anti-CD5 (53–7.3), anti-CD8a (53–6.7), anti-CD11b (MI/70), anti-CD24 (MI/69), anti-CD28 (E18), anti-CD44 (IM7), anti-CD62L (MEL-14), anti-CD69 (H1.2F3), anti-IA/IE (M5/114.15.2), anti-TCRβ (H57-597), anti-TCRVα2 (B20.1), anti-TCRVβ5.1/5.2 (MR9-4) all from BD; anti-CD24 (M1/69), anti-CD25 (PC61.5), anti-CD71 (R17217), anti-CD98 (RL388), anti-CD161c (PK136), anti-CD272 (6A6), anti-CD278 (15P9), anti-CD279 (29F.1A12) all from BioLegend; and anti-FOXP3 (FJK-16s) and anti-CD45 (30-F11) from eBioscience. Stained cells were analyzed using BD FACSymphony A3 Cell Analyzer and BD FACSDiva software. Cell viability was evaluated using DAPI (Life Technologies) or Aqua Dead (Molecular Probes) stains. NKT cells were identified using APC-labeled CD1d tetramers complexed to PBS-57, an analog of α-galactosylceramide, and were provided by the National Institutes of Health (NIH) Tetramer Core Facility. Jurkat T cells were analyzed using anti-CD3 (OKT3), anti-CD28 (CD28.2), and anti-CD69 (FN50), all from BD.

### Mouse T cell proliferation and cytokine production

CD4^+^ and CD8^+^ T cells were purified by immunomagnetic negative selection using CD4 or CD8 EasySep Mouse T Cell Isolation Kit (STEMCELL Technologies) and then stimulated with plate-bound anti-CD3 (145-2C11; Exbio) and soluble anti-CD28 (37–51; Exbio) antibodies or with phorbol 12-myristate 13-acetate and ionomycin. CellTiter-Glo Luminescent Cell Viability Assay (Promega) was used for assessing T cell proliferation. It is based on quantification of the ATP present in the medium and is directly proportional to the number of living cells in the well. At the specified time of culture, 100 μl of CellTiter-Glo reagent (Promega) was added directly to each well and the ATP content measured using a Victor2 luminometer (Wallac; PerkinElmer Life Science). IL-2 and IFN-γ production was measured using BD Cytometric Bead Array.

### Antigen-induced OT-I T cell proliferation and IL-2 production

Naive CD8^+^ T cells were purified from OT-I, OT-I *Carmil*2^−/−^, and OT-I *Carmil2*^QE^ mice by immunomagnetic negative selection using EasySep Mouse Naive CD8^+^ T Cell Isolation Kit (STEMCELL Technologies). Purified T cells were stimulated with irradiated H-2 K^b^–positive spleen cells isolated from T cell–deficient *Cd3ε*^Δ5/Δ5^ or *Cd3ε*^Δ5/Δ5^*Cd80*^−/−^*Cd86*^−/−^ mice and pulsed for 2 h with agonist (N4) and weak agonist (Q4 and T4) OVA peptides. After 48 h of culture, T cell proliferation was assessed with CellTiter-Glo Luminescent Cell Viability Assay (Promega) and IL-2 and IFN-γ production using BD Cytometric Bead Array.

### In vitro suppressive capacity of T_reg_ cells

Single-cell suspensions from spleen and LN of WT, *Carmil2*^QE^, and *Carmil2*^QE^*Cd28*^−/−^ mice were enriched for naive CD4^+^ T cells by negative CD4 isolation (Invitrogen). CD4^+^ CD25^+^ T_reg_ cells from WT, *Carmil2*^QE^, and *Carmil2*^QE^*Cd28*^−/−^ mice and CD4^+^ T_conv_ cells from WT mice were sorted using BD FACSAria III Cell Sorter (BD Biosciences), giving a cell purity >98%. CTV-labeled CD4^+^ CD25^−^ WT responder T cells (5 × 10^4^ cells per well) were cultured in 96-well U-bottom plates with or without sorted T_reg_ cells from WT, *Carmil2*^QE^, and *Carmil2*^QE^*Cd28*^−/−^ mice at responder-to-T_reg_ cell ratios of 1:0, 1:1, 1:2, 2:1, 4:4, 8:1, and 16:1 in the presence of anti-CD3 plus anti-CD28–coated beads (Dynabeads Mouse T-Activator CD3/CD28) at a ratio of two cells for one bead. Cells were collected after 72 h of culture, and proliferation of CTV-labeled CD4^+^ T_conv_ cells was evaluated by flow cytometry by assessing relative CTV dilution.

### Tumor cells

A cyclooxygenase-deficient (Ptgs1/Ptgs2^−/−^) variant of the BRAF^V600E^ mouse melanoma tumor (BRAF^V600E^ Ptgs1/Ptgs2^−/−^ [Zelenay et al., 2015]) was provided by R. Marais (Cancer Research Manchester Institute, Manchester, UK) via C. Reis e Sousa (Francis Crick Institute, London, UK). The MC38-OVA mouse colon adenocarcinoma cell line was provided by J.P. Böttcher (Technical University of Munich, Munich, Germany) (Lacher et al., 2024).

### Ectopic tumor cell inoculation

BRAF^V600E^*Ptgs1*/*Ptgs2*^−/−^ and MC38-OVA tumor cells were harvested by trypsinization, and washed three times with PBS, and 1 × 10^5^ (BRAF^V600E^*Ptgs1*/*Ptgs2*^−/−^) or 5 × 10^5^ (MC38-OVA) cells were injected subcutaneously into the flank of recipient mice in 100 μl of endotoxin-free PBS. The tumor size was quantified as the mean of the longest diameter and its perpendicular. Tumor size volume was calculated by measuring the length and width of a tumor using calipers, and then inputting the values in the equation V=0.5×L×W2, where V is the tumor volume, L is the tumor length, and W is the tumor width. Mice were euthanized when they met physical euthanasia criteria as recommended by national and European laws for laboratory animal welfare and experimentation.

### Adoptive T cell transfer

Naive CD8^+^ T cells were purified from OT-I and OT-I *Carmil2*^QE^ mice as indicated above, and 2 × 10^6^ cells were i.v. injected into B6 mice 6 days after MC38-OVA tumor cell transplantation.

### Anti-PD-1 treatment

MC38-OVA tumor-bearing mice were i.v. injected 6 days after tumor inoculation and then every 3 days with either 200 μg of anti-PD-1 mAb (clone RMP1-14, InVivoPlus anti-mouse PD-1; BioXcell) or 200 μg of an isotype control (InVivoPlus rat IgG2a anti-trinitrophenol; BioXcell).

### Isolation and analysis of myeloid and lymphoid cells from the tumor microenvironment

To isolate cells from tumor mass, tumors were cut into small pieces and transferred to a gentleMACS C tube (Miltenyi Biotec) in 4 ml of RPMI medium supplemented with 5% FCS containing 0.8 mg/ml collagenase 4 (Worthington) and 0.2 mg/ml DNase I (Sigma-Aldrich). The tissue was digested and homogenized using gentleMACS Octo Dissociator and the program suggested by the supplier. The digestion was stopped by the addition of EDTA 0.5 M (5 mM final). Cell suspensions were then filtered through a 100-µm cell strainer prior to flow cytometric analysis.

### Cell lines

The Jurkat human leukemic T cell line and Raji lymphoblastoid B cell line originated from the American Type Culture Collection and were provided by A. Weiss (University of California, San Francisco, San Francisco, CA, USA).

### Generation of Jurkat knock-in cells expressing OST-tagged form of CARMIL2, CARMIL2^Q539E^, and CARMIL2^Q539D^ proteins

A CRISPR-Cas9-based knock-in approach based on a gene trapping strategy permitted to readily introduce biallelic mutations in independent clones of Jurkat T cells (Celis-Gutierrez et al., 2019). We used it to introduce a Twin-Strep-Tag at the C terminus of WT CARMIL2 molecules (CARMIL2^OST^) and in a second knock-in step to obtain mutant CARMIL2^OST^ molecules that contain the Q539E mutation. Using the CRISPOR algorithm we designed two sgRNA permitting to introduce two double-strand breaks (DSB) at the 3′ end of exon 18 of the human *CARMIL2* gene. The following pairs of sgRNA-specifying oligonucleotide sequences were synthesized: Pair 1: 5′-CAC​CGT​TGG​AAG​GAA​CTT​CAA​CGT​C-3′ and 5′-AAA​CGA​CGT​TGA​AGT​TCC​TTC​CAA​C-3′; and Pair 2: 5′-CAC​CGG​GTG​GGG​GCT​CAC​TTG​CAC-3′ and 5′-*AAA​C*GTG​CAA​GTG​AGC​CCC​CAC​CC-3′. Each pair of oligonucleotides contained CACC and AACC overhangs for cloning into *BbsI* sites of plasmid pX330 (pSpCas9; plasmid ID 42230; Addgene), and a G-C base pair (italics) was added at the 5′ end of the guide sequence for T7 transcription. A 4932-bp-long double-stranded DNA (dsDNA) HDR template denoted Carmil2_exons_19-39_OST_P2A_Neo^R^ was designed and purchased from Integrated DNA Technologies as a plasmid vector. It contained (1) a 500-bp-long sequence homologous to the sequence flanking the 5′ end of the first DSB to be introduced in exon 19 (5′ homology arm), (2) a 2,617-bp-long cDNA coding for exons 19 to 39 of the human *CARMIL2* gene, (3) an in-frame 90-bp-long OST tag sequence (Junttila et al., 2005) flanked on both sides by a Gly-Ser-Gly spacer, (4) a 57-bp-long sequence coding for the self-cleaving P2A peptide, (4) a Met (start) codon, (5) a translatable loxP511 sequence, (6) a neomycin resistance gene (Neo^R^)–containing cassette, (7) a stop codon, (8) a loxP511 sequence, (9) a synthetic intron, (10) a polyA sequence, and (11) a 500-bp-long sequence homologous to the sequence flanking the 3′ end of the second DSB to be introduced in exon 20 (3′ homology arm). To prevent CRISPR-Cas9 cleavage of the edited allele, silent mutation destroying the PAM sequences present in the genomic DNA was introduced into the Carmil2_exons_19-39_OST_P2A_Neo^R^ HDR dsDNA template. Jurkat T cells were nucleofected using the Cell Line Nucleofector Kit V program I-010 for Nucleofector II with 2.5 μg of linearized Carmil2_exons_19-39_OST_P2A_Neo^R^ HDR dsDNA template, and 5 μg of pX330-sgRNA Pair 1 and 5 μg of pX330-sgRNA Pair 2 plasmids. Cells were allowed to recover for 48 h and then subjected to G418 selection (2 mg/ml). After 72 h of selection, cells were cloned by limiting dilution and each resulting clone was screened for proper gene editing using PCR and genomic DNA sequencing. Proper knock-in of the Carmil2_exons_19-39_OST_P2A_Neo^R^ cassette allows the expression of the NeoR gene under the control of the *Carmil2* gene. Owing to the high cutting efficiency of CRISPR-Cas9, most of the Jurkat T cell clones that grow following selection in the presence of 2 mg/ml neomycin had the intended biallelic insertion.

### Jurkat T cell stimulation

WT, CARMIL2^OST^, and CARMIL2^QE-OST^ Jurkat T cells (1 × 10^5^) were stimulated by coculture with Raji cells (0.5 × 10^5^) in the presence or absence of the SEE superantigen (Toxin Technology). Following 24 h of stimulation, IL-2 production was measured by ELISA (R&D Systems), and CD69 induction was measured by flow cytometry using anti-CD69 (FN50 from BioLegend).

### Biochemical analysis of Jurkat T cells

WT, *CARMIL2*^OST^, and *CARMIL2*^QE-OST^ Jurkat T cells (60–80 × 10^6^) were incubated with a mouse IgG2a anti-CD3 (5 µg/ml; HIT3a from BD Bioscience) and a mouse IgG1 anti-CD28 (5 µg/ml; CD28.2; BioLegend) for 15 min on ice, followed by one round of washing at 4°C. Cells were then incubated at 37°C for 5 min and either left untreated or stimulated by cross-linking the bound anti-CD3 and anti-CD28 antibodies with purified rat anti-mouse IgG1 (2.5 µg/ml; #553440 from BD Bioscience) and purified rat anti-mouse IgG2a (2.5 µg/ml; #553387; BD Bioscience) for 2 and 5 min at 37°C. The stimulation was stopped by the addition of twice-concentrated lysis buffer (100 mM Tris, pH 7.5, 4 mM EDTA, 300 mM NaCl, 50 mM NaF, 2 mM sodium orthovanadate, and 2% NP-40) supplemented with protease inhibitors. After 10 min of incubation on ice, cell lysates were centrifuged at 21,000 *g* for 8 min at 4°C. Postnuclear cell lysates were either directly used for immunoblot analysis or affinity-purified on Strep-Tactin Sepharose beads. The following antibodies were used for immunoblot analysis: anti-ZAP70 (2705; Cell Signaling Technology), anti-CARMIL2 (EM-53; Exbio Praha), and anti-CARD11 (1D1; Cell Signaling Technology). Unprocessed western blots corresponding to the biochemical analysis of Jurkat cells are shown in SourceData F1.

### Biochemical analysis of mouse CD4^+^ and CD8^+^ T cells

WT, *Carmil2*^OST^, and *Carmil2*^QE-OST^ CD4^+^ mouse T cells were incubated with 0.2 μg per 10^6^ cells of rat anti-CD3 (17A2 from eBioscience) or hamster anti-CD3 (145-2C11; Exbio) in the presence or absence of 0.2 μg per 10^6^ cells of rat anti-CD28 (MAB4832 from R&D Systems) for 15 min on ice, followed by one round of washing at 4°C. Cells were then incubated at 37°C for 5 min and left unstimulated or stimulated by cross-linking the bound anti-CD3 and anti-CD28 antibodies with 0.4 μg per 10^6^ cells of purified F(ab′)_2_ goat anti-rat IgG antibody (112-006-062; Jackson ImmunoResearch) for 2 and 5 min at 37°C. The stimulation was stopped by the addition of twice-concentrated lysis buffer (100 mM Tris, pH 7.5, 300 mM NaCl, 2 mM EDTA, 50 nM NaF, 2 mM sodium orthovanadate, and 2% NP-40) supplemented with protease inhibitors. After 10 min of incubation on ice, cell lysates were centrifuged at 21,000 *g* for 8 min at 4°C. Equal amounts of proteins from postnuclear cell lysates were either directly used for whole-cell lysate immunoblot analysis or affinity-purified on Strep-Tactin Sepharose beads. The following antibodies were used for immunoblot analysis: anti-ZAP-70 (2705 from Cell Signaling Technology), anti-CARMIL2 (EM-53 from Exbio Praha), and anti-CARD11 (1D12 from Cell Signaling Technology). For pervanadate stimulation, a pervanadate stock solution was made by mixing 7.6 vol of water with 1.9 vol of hydrogen peroxide (10 mM final concentration) and with 0.5 vo of sodium orthovanadate (100 μM final concentration), followed by incubation for 15 min at 20°C before addition to T cells. Short-term expanded WT, OTI *Carmil2*^WT^, and OTI *Carmil2*^QE^ CD8^+^ T cells and WT, *Carmil2*^QE^, and *Carmil2*^QE^*Cd28*^−/−^ CD4^+^ T cells were incubated with pervanadate for 2 and 5 min, and stimulation was stopped by the addition of twice-concentrated lysis buffer (see above). Cell lysates were prepared as described above for CD4^+^ mouse T cells. CARMIL2 and CARMIL2^QE^ proteins were immunoprecipitated with a mouse anti-CARMIL2 (EM-53), separated by electrophoresis through 8% SDS–acrylamide gels. Equal amounts of proteins from postnuclear cell lysates were either directly used for whole-cell lysate immunoblot analysis or immunoprecipitated prior to immunoblot analysis. The following antibodies were used for immunoblot analysis: mouse anti-CARMIL2 (EM-53), monoclonal rabbit anti-CARD11 (2D11 from Cell Signaling Technology), anti-ZAP-70 (2705 from Cell Signaling Technology), anti-VAV1 (2505 from Cell Signaling Technology), anti-pY28-SLP76 (558367 from BD Biosciences), anti-pERK1-2 (9106 from Cell Signaling Technology), and anti-pY171-LAT (3581S; from Cell Signaling Technology). Unprocessed western blots corresponding to the biochemical analysis of mouse CD4^+^ and CD8^+^ T cells are shown in Source Data F2.

### Immunoblot quantitation

Protein band areas from the immunoblots were defined as region of interest and quantified using ImageJ software (https://imagej.net/ij/). In the case of affinity purification followed by immunoblots, the CARD11-CARMIL2 ratio was calculated for each condition and normalized to the highest ratio value to give CARD11-CARMIL2 relative units. In the case of immunoblots of the total cell lysate, an anti-ZAP70 was used as a normalization control. The CARMIL2-ZAP70 and CARD11-ZAP70 ratios were calculated for each condition and normalized to the highest ratio value and then expressed in relative units.

### Stimulation and lysis of short-term expanded mouse CD4^+^ T cells prior to AP-MS analysis

Purified CD4^+^ T cells from *Carmil2*^OST^ and *Carmil2*^QE-OST^ mice were briefly expanded for 96 h (Voisinne et al., 2019), rested down, and left untreated or treated for 2, 5, and 10 min with pervanadate (see above). Stimulation was stopped by the addition of a twice-concentrated lysis buffer (100 mM Tris, pH 7.5, 300 mM NaCl, 2 mM EDTA, 50 nM NaF, 2 mM sodium orthovanadate, 2% NP-40) supplemented with protease inhibitors. After 10 min of incubation on ice, cell lysates were centrifuged at 21,000 *g* for 5 min at 4°C and then used for affinity purification.

### Affinity purification of OST-tagged protein complexes

Equal amounts of postnuclear lysates were incubated with Strep-Tactin Sepharose beads (IBA GmbH) for 1.5 h at 4°C on a rotary wheel. Beads were then washed five times with 1 ml of lysis buffer 1× in the absence of detergent and of protease and phosphatase inhibitors. Proteins were eluted from the Strep-Tactin Sepharose beads with 2.5 mM D-biotin, a ligand that binds to Strep-Tactin with a higher affinity than the OST sequence does.

### Tandem MS analysis

Following affinity purification, protein samples were air-dried in a SpeedVac concentrator, and reconstituted in 1% sodium deoxycholate, 50 mM Tris, pH 8. Cysteine residues were reduced and alkylated using 10 mM TCEP and 40 mM chloroacetamide for 5 min at 95°C, and samples were then processed for trypsin digestion using the SP3 method (Hughes et al., 2019) on Sera-Mag carboxylate-modified magnetic beads (Cytiva). Resulting tryptic peptides were resuspended in 17 μl of 2% acetonitrile and 0.05% trifluoroacetic acid and analyzed by nano-liquid chromatography coupled to tandem MS, using an UltiMate 3000 system (NCS-3500RS Nano/Cap System; Thermo Fisher Scientific) coupled to an Orbitrap Q Exactive mass spectrometer (model Q Exactive Plus; Thermo Fisher Scientific). Five microliters of each sample was loaded on a C18 precolumn (300 µm inner diameter × 5 mm, Thermo Fisher Scientific) in a solvent made of 2% acetonitrile and 0.05% trifluoroacetic acid, at a flow rate of 20 μl/min. After 5 min of desalting, the precolumn was switched online with the analytical C18 column (75 µm inner diameter × 50 cm, Acclaim PepMap C18, 2 µM, Thermo Fisher Scientific, or in-house packed with 3 µm Reprosil C18) equilibrated in 95% solvent A (5% acetonitrile, 0.2% formic acid) and 5% solvent B (80% acetonitrile, 0.2% formic acid). Peptides were eluted using a 5–50% gradient of solvent B over 60 min at a flow rate of 300 nl/min. The mass spectrometer was operated in data-dependent acquisition mode with Xcalibur software. MS survey scans were acquired with a resolution of 70,000 and an AGC target of 3e6. The 10 most intense ions were selected for fragmentation by high-energy collision–induced dissociation, and the resulting fragments were analyzed at a resolution of 17,500 using an AGC target of 1e5 and a maximum fill time of 50 ms, respectively. Dynamic exclusion was used within 30 s to prevent repetitive selection of the same peptide.

### Protein identification and quantification for interaction proteomics

Raw MS files were processed with MaxQuant software (version 1.5.2.8) for database search with the Andromeda search engine and quantitative analysis. Data were searched against *Mus musculus* entries of the UniProt KB protein database (release UniProtKB/Swiss-Prot+TrEMBL 2017_01, 89297 entries including isoforms), plus the One-Strep-tag peptide sequence, and the set of common contaminants were provided by MaxQuant. Carbamidomethylation of cysteines was set as a fixed modification, whereas oxidation of methionine, protein N-terminal acetylation, and phosphorylation of serine, threonine, and tyrosine were set as variable modifications. Specificity of trypsin digestion was set for cleavage after K or R, and two missed trypsin cleavage sites were allowed. The precursor mass tolerance was set to 20 ppm for the first search and 4.5 ppm for the main Andromeda database search. The mass tolerance in tandem MS mode was set to 0.5 Da. Minimum peptide length was set to 7 amino acids, and minimum number of unique or razor peptides was set to 1 for validation. The I = L option of MaxQuant was enabled to avoid erroneous assignation of undistinguishable peptides belonging to very homologous proteins. Andromeda results were validated by the target decoy approach using a reverse database, with a false discovery rate set at 1% at both PSM (peptide sequence match) and protein levels. For label-free relative quantification of the samples, the match between runs option of MaxQuant was enabled with a match time window of 1 min, to allow cross-assignment of MS features detected in the different runs, after alignment of the runs with a time window of 20 min. Protein quantification was based on unique and razor peptides. The minimum ratio count was set to 1 for label-free quantification (LFQ) calculation, and computation of the iBAQ metric was also enabled.

### Calculation of interaction stoichiometries

For a given condition of stimulation (represented by the time of stimulation t, with t = 0 s corresponding to the nonstimulated condition), the stoichiometry of the interaction between a prey x and a given bait (denoted bait < x) was computed using

$$S_{\text{bait}<x}\left(t\right)=<I_{\text{OST},x}\left(t\right)>/<I_{\text{OST},\text{bait}}\left(t\right)>*N_{\text{pep},\text{bait}}/N_{\text{pep},x}$$

<I_OST,x_> corresponds to the normalized intensity of protein x in OST-tagged samples and stimulation time t averaged (geometric mean) across all biological replicates, and N_pep_ corresponds to the number of tryptic peptides theoretically observables as estimated from iBAQ values. We also computed stoichiometries independently for each biological replicate and used those values to quantify the regulation of bait–prey association following TCR engagement. For a given condition of stimulation, log-transformed stoichiometries were compared with that of the nonstimulated condition using a two-tailed Welch *t* test. We selected preys whose interaction stoichiometry changed at least twofold with a P value below 0.05 in at least one condition of stimulation as compared to the nonstimulated condition.

### Online supplemental material

Fig. S1 shows the schematic structure of the CARMIL proteins produced by WT mice and *Carmil2*^QE^, *Carmil2*^OST^, and *Carmil2*^QE-OST^ gene-targeted mice. Fig. S2 shows the expression profile of cell surface markers on CD4^+^ and CD8^+^ T cells from WT, *Carmil2*^OST^, *Carmil2*^QE-OST^, OT-I *Carmil2*, OT-I *Carmil2*^QE^, and *Carmil2*^QE^*Cd28*^−/−^mice. Fig. S3 shows that OT-I T cells develop normally in the presence of CARMIL2^QE^ molecules. Fig. S4 shows a model summarizing the CD28-dependent traits induced by the CD28 signaling branches dependent on or independent from CARMIL2-CARD11. Fig. S5 shows that T cells developing in *Carmil2*^QE^ and *Carmil2*^QE-OST^ mice had a similar phenotype. Table S1 shows the criteria used to define whether CARMIL2-CARD11-driven signals are necessary and sufficient to trigger a given CD28-dependent trait. Table S2 shows the list of the proteins interacting with both CARMIL2 and CARMIL2^QE^ molecules in mouse CD4^+^ T cells after 2 min of activation. Table S3 shows the single-strand DNA HDR template sequences used in the present study. Table S4 shows the sgRNA sequences used in the present study. Data S1 is related to Fig. 9 and shows the interactome dataset of CD4^+^ T cells isolated from *Carmil*2 and *Carmil*2^QE^ mice. To facilitate their comparison, the data corresponding to *Carmil*2 (WT) and *Carmil*2^QE^ (QE) CD4^+^ T cells are shown side by side. Data S2 is related to Fig. 9 and shows the correlation analysis permitting to define the CARMIL2 interactors that show a temporal profile of interaction stoichiometry similar to that of the CARMIL2-CARD11 and CARMIL2^QE^-CARD11 interactions. SourceData F1 and SourceData F2 show the unprocessed western blots corresponding to Figs. 1 and 2.

## Supplementary Material

Table S1 shows criteria used to define whether CARMIL2-CARD11-driven signals are necessary and sufficient to trigger a given CD28-dependent trait.

Table S2 shows the list of the proteins interacting with both CARMIL2 and CARMIL2^QE^ molecules in mouse CD4^+^ T cells after 2 min of activation.

Table S3 shows single-strand DNA HDR template sequences.

Table S4 shows sgRNA sequences.

Data S1 is related to Fig. 9 and shows the interactome dataset of CD4^+^ T cells isolated from *Carmil*2 and *Carmil*2^QE^ mice.

Data S2 is related to Fig. 9 and shows the correlation analysis permitting to define the CARMIL2 interactors that show a temporal profile of interaction stoichiometry similar to that of the CARMIL2-CARD11 and CARMIL2^QE^-CARD11 interactions.

SourceData F1 is the source file for Fig. 1.

SourceData F2 is the source file for Fig. 2.

## Data Availability

The mass spectrometry proteomics data underlying Data S1 and S2 are openly available in the ProteomeXchange Consortium at the PRIDE partner repository (https://www.ebi.ac.uk/pride) with the dataset identifier PXD055893. All other data are available in the article and its supplementary files.
