# Supplementary material for: A CARMIL2 gain-of-function mutation suffices to trigger most CD28 costimulatory functions in vivo
Source: J Exp Med. 2025 May 22;222(8):e20250339. doi: 10.1084/jem.20250339 (PMC12097149; doi:10.1084/jem.20250339)
Supplement: Table S1 — shows criteria used to define whether CARMIL2-CARD11-driven signals are necessary and sufficient to trigger a given CD28-dependent trait. [file jem_20250339_tables1.docx]

**Table S1. Criteria used to define whether CARMIL2-CARD11 driven signals are necessary and sufficient to trigger a given CD28-dependent trait**

**
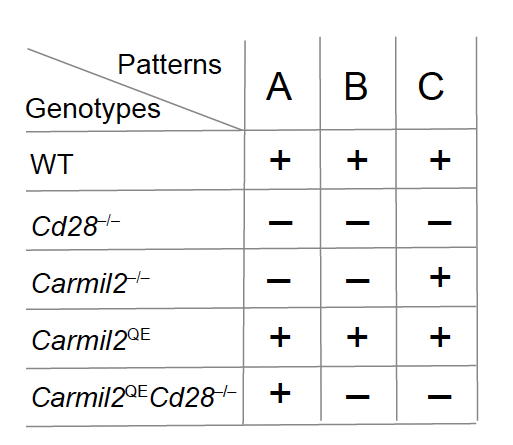
**

The expression of a given CD28-dependent trait among WT, *Cd28*^–/–^, *Carmil2*^–/–^, *Carmil2*^QE^, and *Carmil2*^QE^*Cd28*^–/–^ mice can follow three possible patterns denoted A, B, and C. In pattern A, CARMIL2-CARD11 driven signals are necessary and sufficient to trigger the considered CD28-dependent trait. In pattern B, CARMIL2-CARD11 driven signals are necessary but not sufficient to trigger the considered CD28-dependent trait, suggesting that they need to cooperate with CARMIL2-CARD11 independent CD28 signals to trigger such trait. In pattern C, the considered CD28-dependent trait unfolds in absence of CARMIL2-CARD11 signals.
