# Supplementary material for: A CARMIL2 gain-of-function mutation suffices to trigger most CD28 costimulatory functions in vivo
Source: J Exp Med. 2025 May 22;222(8):e20250339. doi: 10.1084/jem.20250339 (PMC12097149; doi:10.1084/jem.20250339)
Supplement: Table S2 — shows the list of the proteins interacting with both CARMIL2 and CARMIL2QE molecules in mouse CD4+ T cells after 2 min of activation. [file jem_20250339_tables2.docx]

**Table S2. List of the proteins interacting with both CARMIL2 and CARMIL2^QE^ molecules in mouse CD4^+^ T cells after 2 min of activation**

CAPZB

CAPZA2

CARD11

USP9X

PTPRC

VAV1

CD28

INPP4B

CBR1

ANKRD44

CK1-α

PTPN22

GMIP

KIF11

MAP4K1

NCAPD2

TRIM34B

SBF1

JAK1

POLR2B

PTPN6

RAP1A

CAMK2D

FCHO1

ANKHD1

RASGRP2

ACAP1

PPP6R1

USP4

PKC-Θ

In addition to the interacting proteins discussed in the Results section, VAV1, a guanine nucleotide exchange factor previously identified as part of the CD28 interactome (Chen et al., 2022; Skanland and Tasken, 2019)) was also found among the CARMIL2 and CARMIL2^QE^ interactors. Early studies on proteins belonging to the CARMIL family emphasized their ability to regulate the actin-binding activity of capping protein (CP) via their capping protein-interaction motifs (CPI) (Stark et al., 2017). Consistent with that view, the CARMIL2 and CARMIL2^QE^ interactomes comprise, in addition to CAPZB and CAPZA2, several other proteins involved in cytoskeletal remodeling (GMIP) and vesicular transport (FCHO1, KIFL1, ACAP1, KIF11). The role of the remaining proteins found in the CARMIL2- and CARMIL2^QE^ interactomes remains to be elucidated.
