# Supplementary material for: A CARMIL2 gain-of-function mutation suffices to trigger most CD28 costimulatory functions in vivo
Source: J Exp Med. 2025 May 22;222(8):e20250339. doi: 10.1084/jem.20250339 (PMC12097149; doi:10.1084/jem.20250339)
Supplement: Table S3 — shows single-strand DNA HDR template sequences. [file jem_20250339_tables3.docx]

**Table S3. Single strand DNA HDR template sequences**

| **Gene** | **Sequence (5’ to 3’)** |
| --- | --- |
| **OST Tag**  **insertion**  **in *Carmil2***  **exon 1** | TTAATACGACTCACTATAGGGAGGCAGAGGCAGGTGGATTTCTGAGTTCGAGGCCAGCCTGGTCTATACAAAGTGAGCTCCAGGACAGCCCAGGCTACGCAGAGAAACCCTGTCTCGGGGAGAAAAAAAAAAAAAAAAGGGTGCAGCCCAGCCTCTTTTCTTCCTGCCTGTTTTTACAGTGCTGGGATTACAAATAGGTTGCCAGACTCTTAAATTCGTATCTGAGCAGGGCAGAACCTTGGGTGGTGGGTAATTTTTACTGCACAGAGACACAACTGCTCTGGCTTCCTTTGAGTTACTCCAACCTACCCAACCTGTGTCTTGGGATAGATCAAGTTAGTTAGGCCTTTGGCATCCCCTGGAGGGTGGCGTCCCCTGGTGTTTGCGCCCGGGACTACGGGGCAGGGTGGAAGTAGGGCACCAGGCAAGGGGTGAAAAGGCAAGACCCTCTCCCCTACACACCACACCCATGAGTACCCGAGTACGCGGAAGGAAAAGTCCCTGAGCGACAGCAACAGTCAAGATTCGACCATCCTCCCACAACCTCCCACCCGGGGCGAACTCAGCCAACGAAGCCGGGGCGGGGAGGGGCCGGGCCGGGGGCGGCCTGGCAGGAAGAAGCGCGTACCTTCCGCTGCAGGAGGAGCAGGTGGCTGCACTGCTGCCTGGGCCCCTGGCTTCCTGTGTACGCTCCTGCCGGCTGCTTTTTCCCGCCAGAGCTCATTGGGCCGTCCCGGCCCTATGTCCGCATGGAGCCACCCGCAGTTCGAGAAAGGTGGAGGTTCAGGAGGTGGATCGGGAGGTTCGGCATGGAGCCACCCGCAGTTCGAAAAAGGTTCCGGGGCACAGACCCCAGACGACATATCCTGTGAACTGCGAGGTAAGCCCTGGGAGCCCACCTGGCCATGAAGAGCCTGACTCCAAACCAGACCTCTCCTCTCGGCGCCTTAGGGAACTTGACCAGGGGTTTTTGTTCTGGAGGGAGGGCCAGTGCCTAAAAGTCCGCTTCATTTCTTCCTAGCTGATCCACACATGAATACCTGGGCACTCAGTCTAGCACAGACCTTGGGCACAGAGTGGCCCTAATAGAGGAGCCAAAGTCACCCAGTAGACTGTCTTTCCACAGGCGAGATCACCAGGTTCCTGTGGCCCAAGGAGGCGGAGCTGCTGTTGAAAACCTGGCTTCCCCAGGAGGGTGCCGAGCAAAGCCATATCCTGGTATGGTAGCCAGGAGTGAAGCTCAGGACAGTGGCCAGACCCTGCACAAAGAAACTTGATTTCCCTAGAGTATTAGTCTTTTGTCTCCCCCCAGGCACTGCTTCGATGGAGGGCATATTTGCTGCACACCTGCCTTCCCCTGAGGGTGAGTCCCAGGCCGGGCCAACACCCCTTCCCCTTCAGCAAGGGACTGCCTTGCGCAGCTGTCTTCTTTGCCCATGCTGGCTTTTACTTTCCAACAGCAGACAGGAGGGAAGTCTGGGCAGGTCTAGCTCAGGATGCCACAAAATCATGGGCTGTGGAGAGAGGACCTGGGTTCAATTCTCCCACCACCAGGTGGGCTCACAAGTGGTTGGAACTCTAGTTCGAAGGCACGAATTC |
| **Q538E**  **mutation**  ***in Carmil2***  **exon 20** | CAGCAGCCCCTTTCCACAGGGAGACCCTGGACGATGTCCTGCATCGGATAGCTGAGCTAATGCAGGATGACGACTGTGTGAGTTCACAGAGCCCTGTGGGGGGTCCT |
