# Supplementary material for: A CARMIL2 gain-of-function mutation suffices to trigger most CD28 costimulatory functions in vivo
Source: J Exp Med. 2025 May 22;222(8):e20250339. doi: 10.1084/jem.20250339 (PMC12097149; doi:10.1084/jem.20250339)
Supplement: Table S4 — shows sgRNA sequences. [file jem_20250339_tables4.docx]

**Table S4. sgRNA sequences**

| Gene | sgRNA target sequence (5′ to 3′) |
| --- | --- |
| ***Carmil2* exon 1** | CGTCTGGGGTCTGTGCCATA *GGG* |
| ***Carmil2* exon 1** | ATTGGGCCGTCCCGGCCCTA *TGG* |
| ***Carmil2* exon 20** | AGTCGTCATCCTGCATTAGC *TGG* |
