# Supplementary figures and images for: A CARMIL2 gain-of-function mutation suffices to trigger most CD28 costimulatory functions in vivo
Source: J Exp Med. 2025 May 22;222(8):e20250339. doi: 10.1084/jem.20250339 (PMC12097149; doi:10.1084/jem.20250339)

Uncropped blots\_Figure 1

Panel B

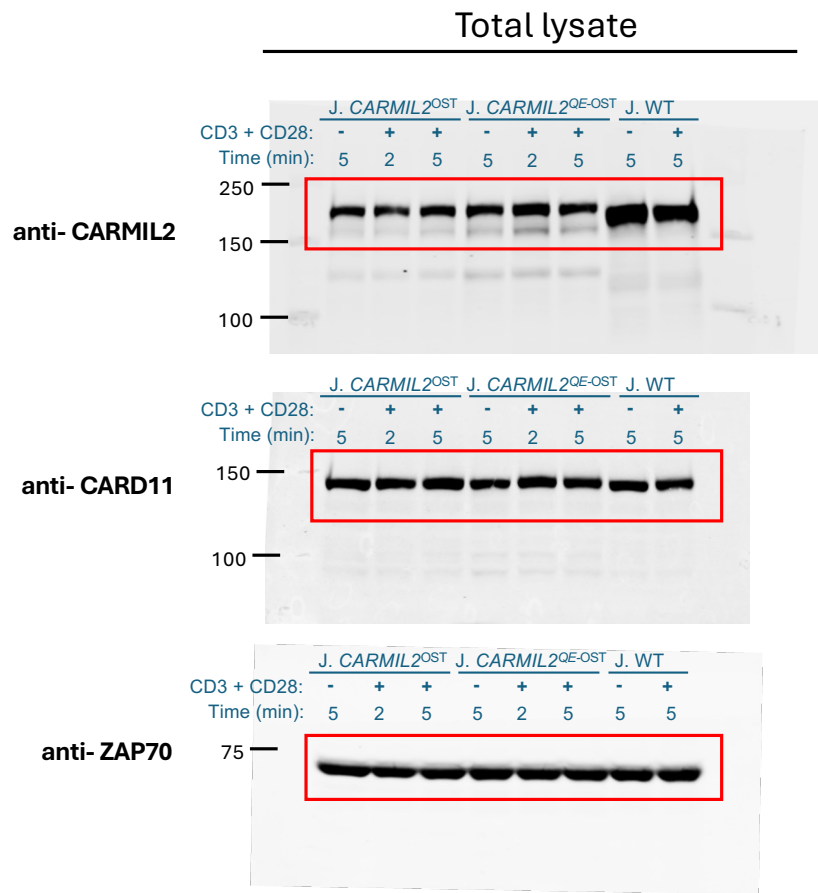

Panel D

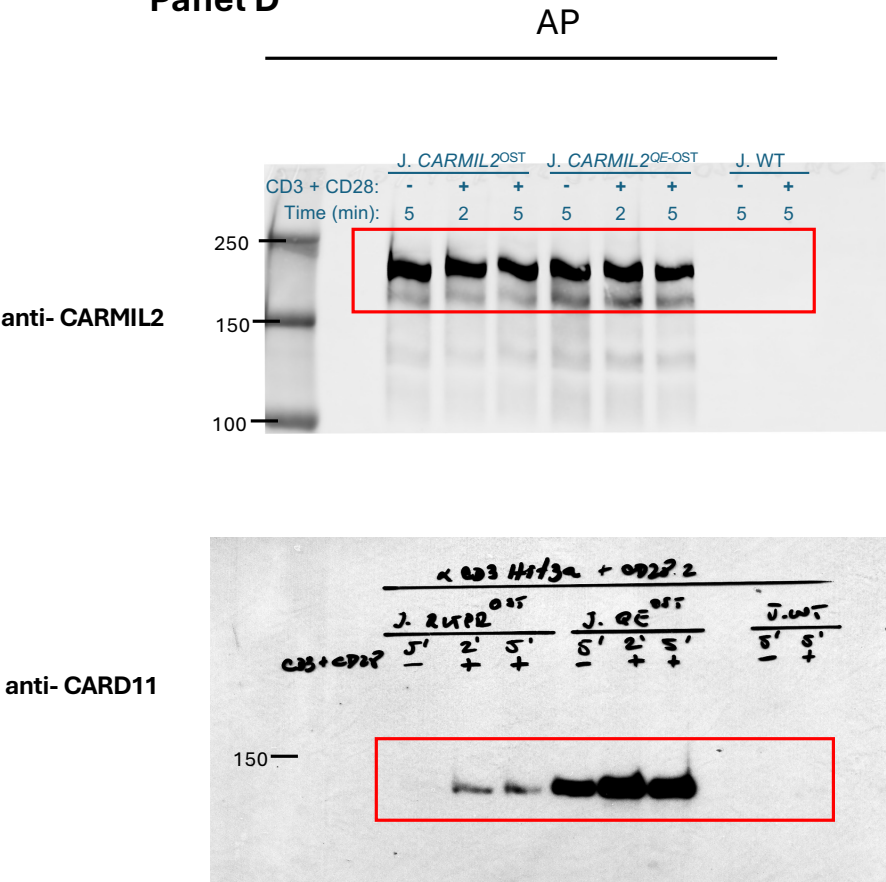

Uncropped blots\_Figure 1

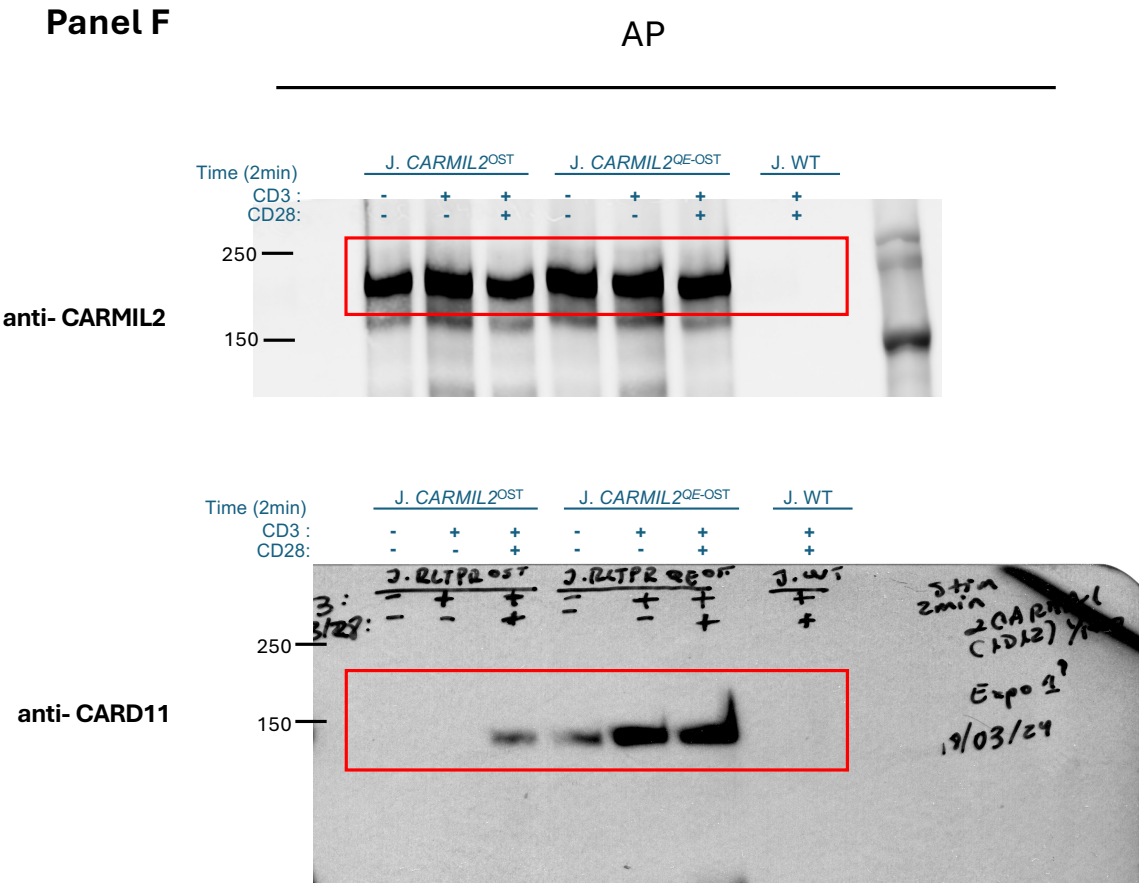

Supplement: SourceData F1 — is the source file for Fig. 1. [file jem_20250339_sourcedataf1.pdf]
