# Supplementary material for: A CARMIL2 gain-of-function mutation suffices to trigger most CD28 costimulatory functions in vivo
Source: J Exp Med. 2025 May 22;222(8):e20250339. doi: 10.1084/jem.20250339 (PMC12097149; doi:10.1084/jem.20250339)

Uncropped blots_Figure 2

Panel A

Total lysate

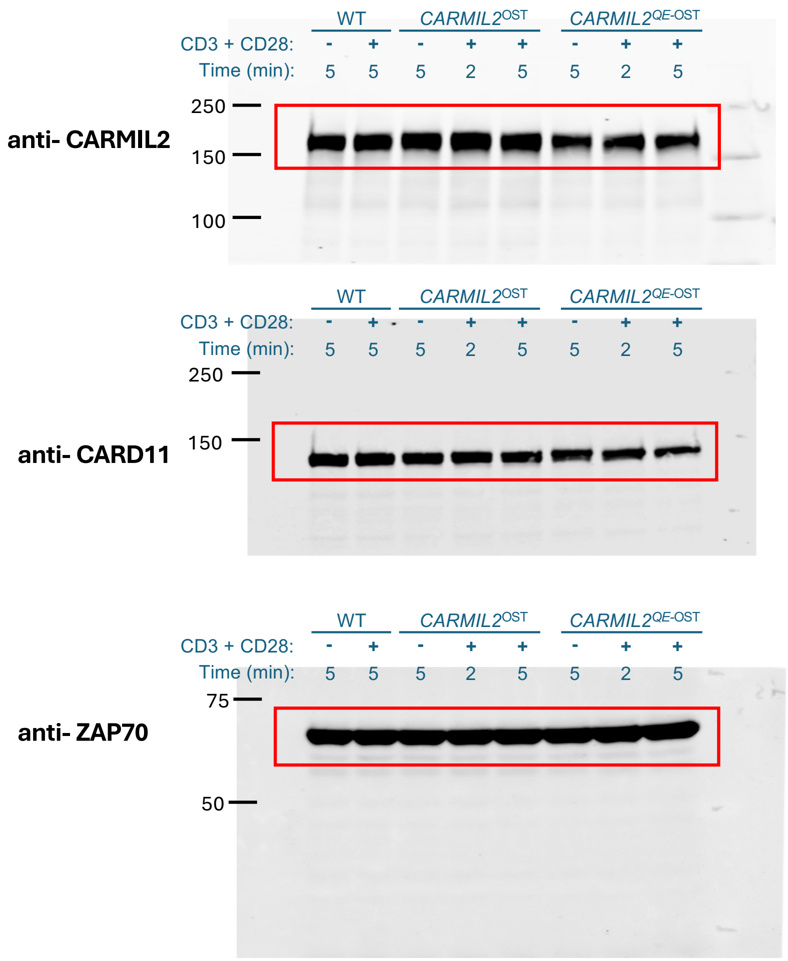

Panel B

AP

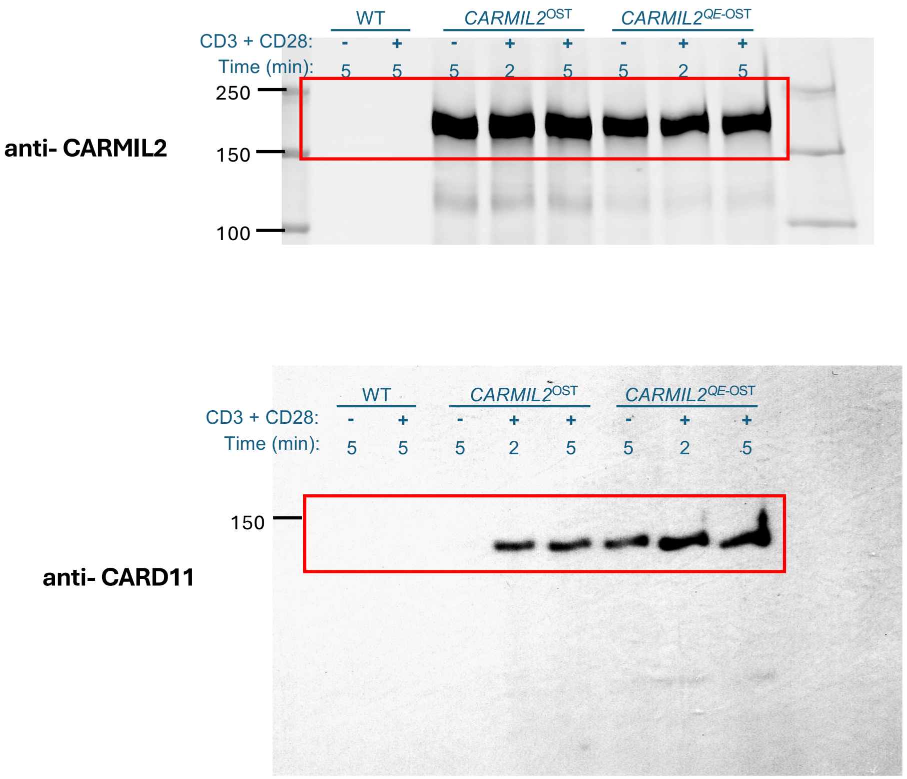

Uncropped blots_Figure 2

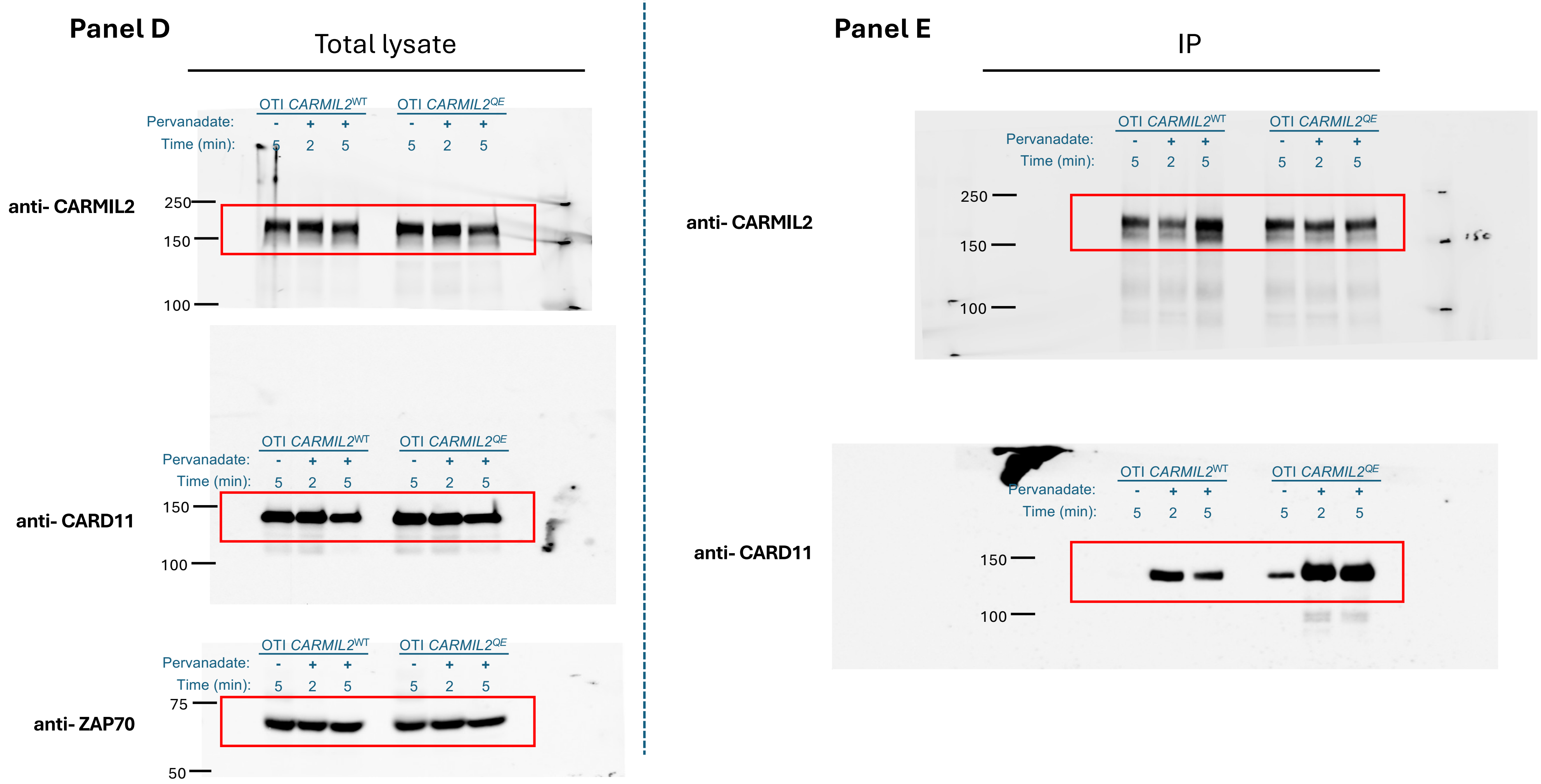

Uncropped blots_Figure 2

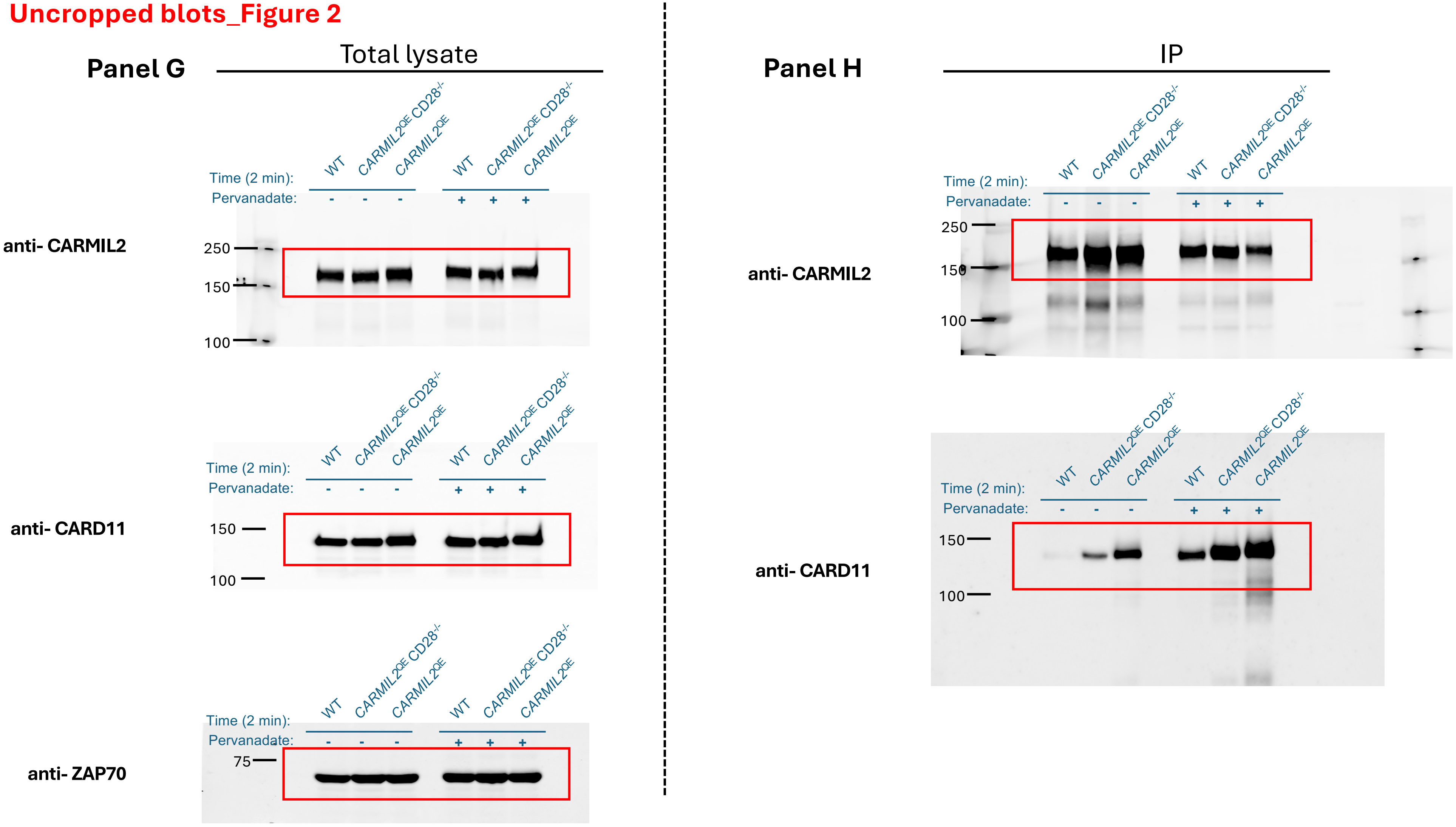

Uncropped blots_Figure 2

Panel J

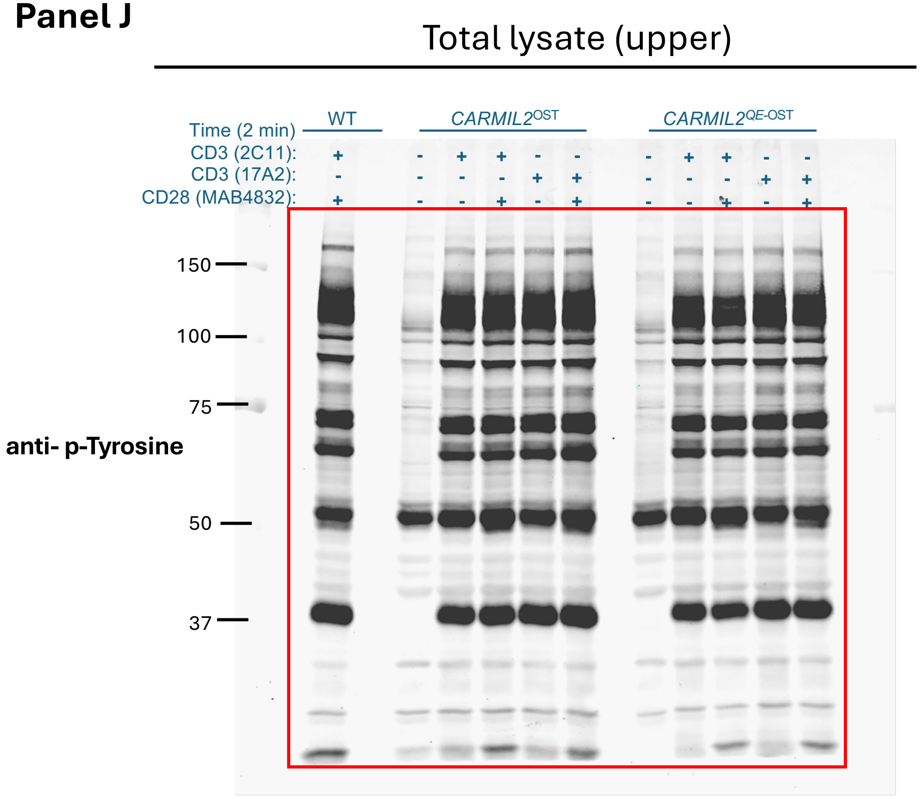

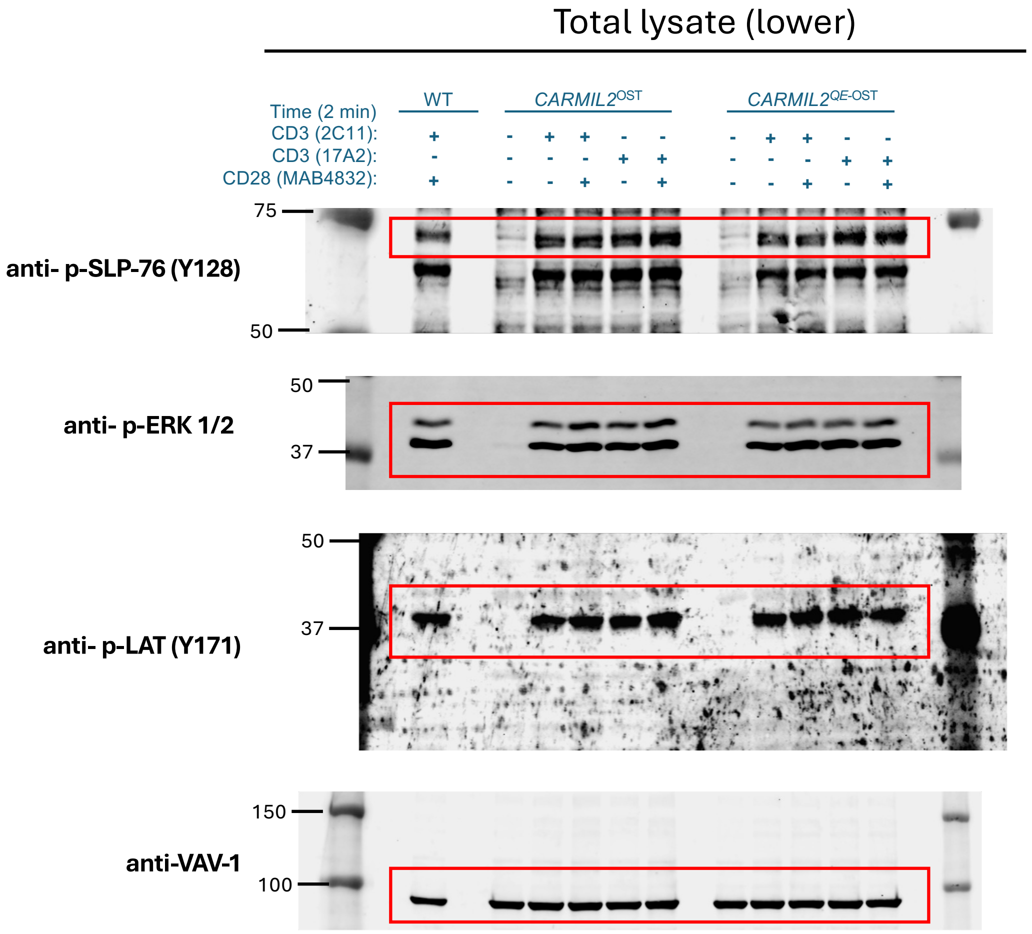

Supplement: SourceData F2 — is the source file for Fig. 2. [file jem_20250339_sourcedataf2.pdf]
